# Introduction of protein vaccine candidate based on AP65, AP33, and α-actinin proteins against *Trichomonas vaginalis* parasite: an immunoinformatics design

**DOI:** 10.1186/s13071-024-06248-y

**Published:** 2024-03-31

**Authors:** Forozan Ghasemi Nezhad, Afsaneh Karmostaji, Parisa Sarkoohi, Behzad Shahbazi, Zahra Gharibi, Batul Negahdari, Khadijeh Ahmadi

**Affiliations:** 1 Student Research Committee, Faculty of Pharmacy, Hormozgan University of Medical Sciences, Bandar Abbas, Iran; 2 Infectious and Tropical Diseases Research Center, Hormozgan Health Institute, Hormozgan University of Medical Sciences, Bandar Abbas, Iran; 3 Department of Pharmacology and Toxicology, Faculty of Pharmacy, Hormozgan University of Medical Sciences, Bandar Abbas, Iran; 4 School of Pharmacy, Semnan University of Medical Sciences, Semnan, Iran

**Keywords:** *Trichomonas vaginalis*, Epitope, Protein, Docking, Molecular dynamics

## Abstract

**Background:**

*Trichomonas vaginalis* is the most common nonviral sexually transmitted disease (STI) worldwide. Vaccination is generally considered to be one of the most effective methods of preventing infectious diseases. Using AP65, AP33 and α-actinin proteins, this research aims to develop a protein vaccine against *Trichomonas vaginalis*.

**Methods:**

Based on the B-cell and T-cell epitope prediction servers, the most antigenic epitopes were selected, and with the necessary evaluations, epitope-rich domains of three proteins, AP65, AP33, and α-actinin, were selected and linked. Subsequently, the ability of the vaccine to interact with toll-like receptors 2 and 4 (TLR2 and TLR4) was assessed. The stability of the interactions was also studied by molecular dynamics for a duration of 100 nanoseconds.

**Results:**

The designed protein consists of 780 amino acids with a molecular weight of 85247.31 daltons. The results of the interaction of the vaccine candidate with TLR2 and TLR4 of the immune system also showed that there are strong interactions between the vaccine candidate protein with TLR2 (-890.7 kcal mol^-1^) and TLR4 (-967.3 kcal mol^-1^). All parameters studied to evaluate the stability of the protein structure and the protein-TLR2 and protein-TLR4 complexes showed that the structure of the vaccine candidate protein is stable alone and in complex with the immune system receptors. Investigation of the ability of the designed protein to induce an immune response using the C-ImmSim web server also showed that the designed protein is capable of stimulating B- and T-cell lymphocytes to produce the necessary cytokines and antibodies against *Trichomonas vaginalis*.

**Conclusions:**

Overall, our vaccine may have potential protection against *Trichomonas vaginalis*. However, for experimental in vivo and in vitro studies, it may be a good vaccine candidate.

**Graphical Abstract:**

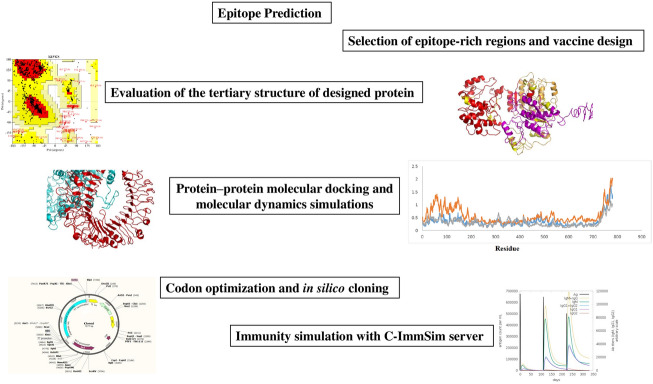

**Supplementary Information:**

The online version contains supplementary material available at 10.1186/s13071-024-06248-y.

## Background

Trichomoniasis is estimated to be the most common nonviral sexually transmitted disease in the world. One hundred fifty-six million cases of trichomoniasis occur annually worldwide. However, in the USA, 6.9 million cases of infection were reported in 2018, with medical costs of $144 million, according to the Centers for Disease Control and Prevention (CDC) [1]. The most widely used and most effective clinical treatment for trichomoniasis is the drug metronidazole. However, the gradual increase of strains of *Trichomonas vaginalis* resistant to this drug, as well as issues such as mutagenicity in bacteria, carcinogenicity in mice, the ability to cross the placenta, and the possibility of teratogenicity in the fetus, do not present metronidazole as a suitable drug for trichomoniasis [2, 3]. Some researchers have suggested that DNA vaccines or recombinant antigens can effectively stimulate immune responses against parasitic protozoa. Therefore, vaccination can be an optimal approach to eradicate infectious diseases such as trichomoniasis [4–6]. However, no commercial vaccine against trichomoniasis has been produced to date. AP65 and AP33 belong to the class of proteins designated as adhesion proteins. Research shows that AP51 and AP65 bind to heme and hemoglobin. AP33 plays a key role in the adhesion process of the parasite to the host. Studies have shown that the interaction between AP33 and BNIP3 mediates the adhesion and pathogenicity of *T. vaginalis* to host cells and is the basis for searching for drug targets and designing vaccines for *T. vaginalis* [7]. Recombinant AP33 protein is highly expressed and an antibody against AP33 was detected by enzyme-linked immunosorbent assay (ELISA) in 78% of 50 patients who were infected with *T. vaginalis*. Different genotypes of *T. vaginalis* are highly homologous in the *ap33* gene. Among different genotypes of *T. vaginalis*, there is a high similarity (100–98.2%) in the *ap33* gene sequence. All these indicated that AP33 can be used as an excellent vaccine candidate antigen against *T. vaginalis* [8]. In researchers’ efforts to determine what factors play a key role in adhesion, the AP65 protein was identified. In addition, the AP65/BNIP3 interaction causes *T. vaginalis* to adhere to host cells and become pathogenic, and this protein is being introduced as a basis for the prevention and treatment of trichomoniasis [9]. The amoeba-like morphology induced by α-actinin is essential for pathogenicity and phagocytosis [2]. α-Actinin is one of the most common immunogens detected in the serum of women infected with *T. vaginalis* [10]. This protein is conserved in many isolates of *T. vaginalis* and can induce antibodies to different epitopes against different strains. The important role of α-actinin in survival and its high immunogenicity indicate its high potential for the development of a vaccine against *T. vaginalis* [2]. Vaccination is generally considered to be one of the most effective methods for the prevention of infectious diseases. In recent years, significant progress has been made in the design and experimental production of multisubunit polypeptide vaccines [11–13]. Mainly T cell and B cell epitopes are used for these types of vaccines. Antigens of appropriate conformation and function are often required to induce functional immune responses [14, 15]. *T. vaginalis*-specific antibody responses and T cell-mediated immune responses may influence the outcome of the infection in terms of parasite clearance, persistence, or pathological responses [16]. Since *T. vaginalis* is an extracellular pathogen, B cells and antibodies are critical in the immune response to extracellular pathogens, and T cells also play an important role in clearing pathogens and providing immune memory [17, 18]. Innate immunity plays an important role in clearance of pathogen infections and defense against invading microorganisms. *T. vaginalis* infection increases the expression of TLR2 and TLR4 in epithelial cells, which may lead to the initiation of the host immune response against *T. vaginalis* [16, 19, 20]. In addition to T cell epitopes, the prediction of B cell epitopes plays an important role in the design of a vaccine against *T. vaginalis* [8]. Protein subunit vaccines are safer than whole-cell attenuated vaccines, but they often have a lower level of immunogenicity [14]. Antigens with different essential mechanisms and appropriate functions are required to design a suitable vaccine candidate to induce functional immune responses [12].

To design subunit vaccines, it is first necessary to identify effective and key antigens in the target organism and evaluate them in terms of antigenic properties and binding to various host proteins. After the necessary investigations, it was found that the three proteins AP65, AP33, and α-actinin have the necessary structural and immunological features as vaccine candidates against the *T. vaginalis* parasite. In this study, using these three proteins that play important roles in the survival and function of *T.vaginalis*, we attempted to design a multiepitope protein that targets different mechanisms.

To develop a reliable vaccine against *T. vaginalis*, we used reverse vaccinology to find immune epitopes of AP33, AP65, and α-actinin proteins that are essential for *T. vaginalis* function. A recombinant *T. vaginalis* protein vaccine candidate was proposed. Computational methods were used to evaluate the interaction of the recombinant protein with TLR2 and TLR4 to assess the biological activity of the proposed protein. Finally, in silico immune simulations were used to analyze the effect of the protein vaccine candidate on the induction of immune function.

## Methods

### *Retrieval of the AP33, AP65, and α-actinin protein sequences*

The sequences of *T. vaginalis* AP33, AP65, and α-actinin proteins were extracted from UniProtKB (https://www.uniprot.org/). The UniProt website provides an intuitive interface to help find the desired protein and discover protein data [21].

### T cell epitope prediction

Stimulation of humoral and cellular immune responses is determined by Helper T lymphocyte (HTL) epitope prediction, which is essential for the design of prophylactic bacterial vaccines. The RANKPEP 1D sequence-based screening server was used to identify T cell epitopes. This server predicts immunodominant peptides in interaction with MHC molecules using a position-specific scoring matrix (PSSM) [22]. Also, the Immune Epitopes and Analysis Resource (IEDB) server (http://tools.immuneepitope.org/mhcii/) was used to predict HTL epitopes, and epitopes with an IC50 value less than 50 and the lowest percentile ranked as T cell epitopes were selected for vaccine design. DRB1*0101, DRB1*0301, DRB1*0401, DRB1*0701, DRB1*0801, DRB1*1101, DRB1*1301, and DRB1*1501, which cover the HLA variability of more than 95% of the human population worldwide, were considered for the prediction of HLA class II epitopes worldwide [23].

### Prediction of linear B cell epitopes

The common databases BepiPred and Kolaskar and Tongaonkar Antigenicity (http://www.iedb.org/) were used to predict linear B cell epitopes. To analyze the results with more confidence, we used both servers. Finally, the epitopes with the best score, which are located in the protected areas of the protein, were selected. The IEDB database collects experiments that identify and characterize epitopes and epitope-specific immune receptors, along with various other details such as host organism, immune exposures, and inducible immune responses [24].

### Selection of epitope-rich regions and vaccine candidates

To select epitope-rich domains, predicted B cell and T cell epitopes were evaluated, and regions of proteins with high and common B cell and T cell epitopes were selected and used to design the final construct as a vaccine candidate. Finally, the epitope-rich domains of B cell and T cell proteins AP33, AP65, and α-actinin were selected and joined together with EAAAK, EAAAKEAAAK, and GGGGS linkers in various states. The resulting constructs were compared based on physicochemical properties, antigenicity, secondary structure, and tertiary structure.

### Physical and chemical characteristics of the final designed structure

Using EXPASY ProtParam server (http://expasy.org/tools/protparam.html), the physical and chemical properties of the final designed structure, such as the number of amino acids, molecular weight, isoelectric ph (PI), the number of charged amino acids, amino acid composition, estimated half-life, instability index, aliphatic index, and grand average of hydropathicity were investigated [25]. Physicochemical properties reflect the functional and structural characteristics of a protein. To know the role of a protein, a comparative study of physicochemical properties is important [26].

### Investigation of antigenicity, allergenicity and solubility of the designed protein

The VaxiJen v2.0 server with a threshold of 4.0 was used to analyze the designed vaccine candidate antigen. VaxiJen classifies antigens based on automatic cross-covariance (ACC), which transforms protein sequences into uniform vectors of key amino acid features [27]. Allergens are small antigens that typically elicit an IgE antibody response. There are two types of bioinformatics-based allergen prediction. The first approach follows the FAO/WHO Codex Alimentarius guidelines and searches for sequence similarity. The second approach is based on the identification of linear motifs associated with conserved sensitization. AllerTOP is the first unparalleled server for in silico prediction of allergens based on key physicochemical properties of proteins [28]. Using AllerTOP (http://www.ddg-pharmfac.net/AllerTOP/), the allergenicity of the designed protein was evaluated [28]. Also, the protein-sol server (https://protein-sol.manchester.ac.uk/) with a threshold of 0.45 was used to predict the propensity of protein solubility. This server calculates 35 sequence-based features using available data on the solubility of *Escherichia coli* proteins in a cell-free expression system [29].

### Secondary and tertiary structure prediction

Secondary structure prediction of the candidate protein was performed using the Garnier–Osguthorpe–Robson (GOR) server based on amino acid sequence. GOR is based on probability parameters obtained experimentally from known protein tertiary structures solved by x-ray crystallography [30]. The tertiary structure was modeled using I-TASSER (https://zhanggroup.org/I-TASSER/) web server [31].

### Evaluation of the tertiary structure of designed protein

MolProbity (http://molprobity.biochem.duke.edu) [32], ProSA-web (https://prosa.services.came.sbg.ac.at/prosa.php) [33], and SAVES (https://saves.mbi.ucla.edu) [34] servers were used to evaluate predicted 3D structure. Identifying errors in experimental and theoretical models of candidate protein structures is one of the essential issues. The designed structures should be close to the structure of proteins found in nature so that they can be expressed in the cell. To check for possible errors in the designed 3D models, the third structure was evaluated with ProSA-web. The *Z*-score and energy plot are used to evaluate the structure. An error in the 3D structure is indicated by a *Z*-score out of range for native proteins [33]. One of the most widely used protein tertiary structure evaluation servers is MolProbity, which is used to confirm the quality of three-dimensional structures of macromolecules such as proteins, nucleic acids, and complexes. Some of the most important parameters analyzed in this web server are MolProbity score, collision score, and Ramachandran diagram [35]. The calculation of the Ramachandran plot is another important parameter for the evaluation of the third structure. In this study, to calculate the torsion angles of the residues in the candidate protein and to show whether the residues in the distant regions are allowed and favorable, the Ramachandran diagram obtained from PROCHECK of the SAVES web server was used [34].

### Conformational B cell epitope prediction

The prediction of the structural epitopes of the vaccine candidate protein was performed using the ElliPro server. The web server uses a modified version of Thornton’s method, the MODELLER program, and Jmol for antibody epitope prediction. Using a designed protein tertiary structure, the ElliPro server predicts B cell structural epitopes [36].

### Investigating the interaction between recombinant protein with immune system receptors

From the PDB database (https://www.rcsb.org), the crystallographic structures of the receptors TLR2 (PDB ID: 53di) and TLR 4 (PDB ID: 7mlm) were extracted. The ligands and the water molecules were removed from the two structures. Hydrogen atoms and charges were then added to the receptors and designed protein structures with the Dock prep tool using the UCSF Chimera 1.10.1 tool [37].

TLR2 and TLR4 are important receptors of the immune system to fight *Acinetobacter baumannii*. Important amino acids in the active site of TLR2 include Leu317, Ile319, Phe322, Leu324, Phe325, Tyr326, Val348, Phe349, and Pro352 [38]. Other important amino acids in the active site of the TLR4 receptor include Arg434, Ser413, Ser386, Arg380, Lys341, Lys263, and Gln339 [39].

Designed protein interaction with TLR2 and TLR4 of the immune system using the Cluspro2 server (https://cluspro.bu.edu/home.php) [40]. Amino acids involved in the interaction between vaccine candidate and receptors were reviewed using LigPlot+ v.4.5.3 [41]. The figures were generated using PyMOL (the PyMOL Molecular Graphics System, Version 2.0 Schrödinger, LLC.). The PRODIGY web server was also used to predict the binding energy of the protein complexes. This server focuses on prediction of binding affinity in biological complexes and identification of biological interfaces [42].

### Molecular dynamics simulation

The most potent vaccine–receptor complexes with the lowest free energy, the highest number of hydrogen and hydrophobic bonds, and the best affinity of the ligand to the receptor were selected for further investigation. The stability of the complexes and the structure of the designed protein were evaluated by Molecular dynamics (MD) simulation. For this purpose, the GROMACS 2018 software package [43] and the OPLS-AA force field were used. The structure was placed in a triaxial box at a distance of 1 nm from all edges. The system was then neutralized by the addition of a specific concentration of Na^+^ and Cl^−^ ions. The positioning parameters of the protein structure were obtained using GROMACS software. The positioning parameters of the protein structure were analyzed using GROMACS software. The vaccine–receptor complexes were then introduced into a simulation chamber filled with TIP3P water molecules. The energy minimization process for the simulated complexes was divided into two parts: in the first part, the systems were equilibrated using NVT (constant number of particles, volume, and temperature) at 300 K and 100 ps, and in the second part, the system was equilibrated to the temperature and to the desired pressure. NPT (constant particle number, pressure, and temperature) was equilibrated at 300 K and 1.0 bar and 100 ps using a Parrinello–Rahman barostat. To deal with long-range electrostatic charges, the Ewald mesh particle algorithm was used and its no-effect distance was considered to be 10 angstroms. A distance of 1 nm was used to calculate van der Waals interactions. A linear constraint algorithm was used to limit the length of covalent bonds. After applying the necessary balances, 100 ns simulation was used for the selected complexes by molecular docking method and designed protein. The output trajectories were analyzed using root mean square fluctuations (RMSF), root mean square distance (RMSD), radius of gyration (RG), principal component analysis (PCA), and free energy landscapes (FELs) to determine stability and protein structure changes during the simulation. The images related to the snapshots have been created using the UCSF Chimera 1.10.1 tool.

### Simulation of the immune system

An immune simulation study was performed for the assessment of the vaccine’s immunogenicity and immune response profile. Immune response induction for the designed protein was performed by the C-ImmSim simulation server (http://150.146.2.1/C-IMMSIM/index.php). The simulation was performed with default parameters and the time steps were set at 1, 336, and 672. The vaccine was injected in three times. The simulation steps were 1000. The simulation volume was 10 µL, and the random seed was 12,345 by default [44].

### Cloning and optimization

To improve recombinant protein production, codon optimization and evaluation of GC content for nucleotide sequence was performed using the tool (JCat) in *E. coli* (K12-strain). SnapGene version 3.2.1 was used to clone the optimized clone of the designed protein into the pET28a vector and to evaluate double digestion.

## Results

### Genome extraction of AP33, AP65, and α-actinin proteins

The sequences of AP33 (accession number: Q65ZG5), AP65 (accession number: Q27093), and α-actinin (accession number: O96524) proteins were extracted from the UniProtKB database.

### Prediction of B cell epitopes

Prediction of B cell epitopes for all three proteins, AP33, AP65, and α-actinin, was done using Bepipred and IEDB servers (Kolaskar and Tongaonkar) (Additional file 1: Table S1).

### Prediction of T-cell epitopes

IEDB and Rankpep databases were also used to predict T cell epitopes. The allelic group for MHCII alleles DRB1*0101, *0301, *0401, *0701, *0801, *1101, *1301, *1501, which covers the genetic background of most humans, was selected. The most important epitopes with the highest score were selected (Additional file 2: Table S2).

### Selection of epitope-rich regions

The regions of AP33, AP65, and α-actinin proteins with the highest epitope abundance are considered as the target domain for vaccine design to select the domains that make up the vaccine candidate. Finally, nine epitope-rich domains from these three proteins were selected as vaccine candidates, which contain a large number of B cell and T cell epitopes (Table 1).

**Table 1:** Nine epitope-rich domains selected from three proteins AP33, AP65, and α-actinin

Antigen	Position	Antigenic determinant
AP33	169–290 50–115	LTYEAAYATTQAGLGQSTVVGIGGDPFAGQLHTDVIKRFAADPQTEGIILIGE IGGTSEEDAAEWIAKTKLTQEKPVVAFIAGATAPPGKRMGHAGAIVSGGKGTAEGKYKALEAAGVRIAR VHPKKAGKIIAGLPIFKNMKEVVKRTDANASLIFVPAPGAAAACIEAAEAGMGLVVCITEHIPQHD
AP65	60–160 320–370 420–480	KDEQAARIRRQFELMPTPLLKYIFLANEREKNSQSFW RFLFTHPPEETMPILYTPTVGEACQKWATHRQSYRGIYITPEDSGKIKDILRNYPRQDIRCIVV IANLIVDMTVSRGGITKEQAFKNIIMFDHRGMVHAGRKDLYDFNKPYMHDM NPTPKAEATPHDVYLWSNGKALCATGSPFPAEQVNGRKVITAQANNSWIFPAVGYALVTTK
α-actinin	500–580 220–330 740–800 620–665	IAFKEEVLAISGELRERRTQFLAKQAEAPTKR EHVNEIDPIFDGLEKDSLHLRVNHSPTEIRNVYAVTLQHIITELNKIFE EKSVVTQVAEFFHFFASESKIAAMADKIKRTVAIQKQIDELKNTYIEDAKAAI EKMTVEDEKLKADDYEKTIPGIRGKLASVISYNRDIRPEIVDHRAKAMRSWAALVTKC EELTPIYEDLEKDQLHLEITSTPASINIFFENLIAHIDTLVKEIDAAIAAAKGLEISEEEL ENLASLDGFAEKIQALQDPYNELVEFKLNYKVTYTYSDATGELDQA

### Protein design using selected domains and different linkers

**Fig. 1:**
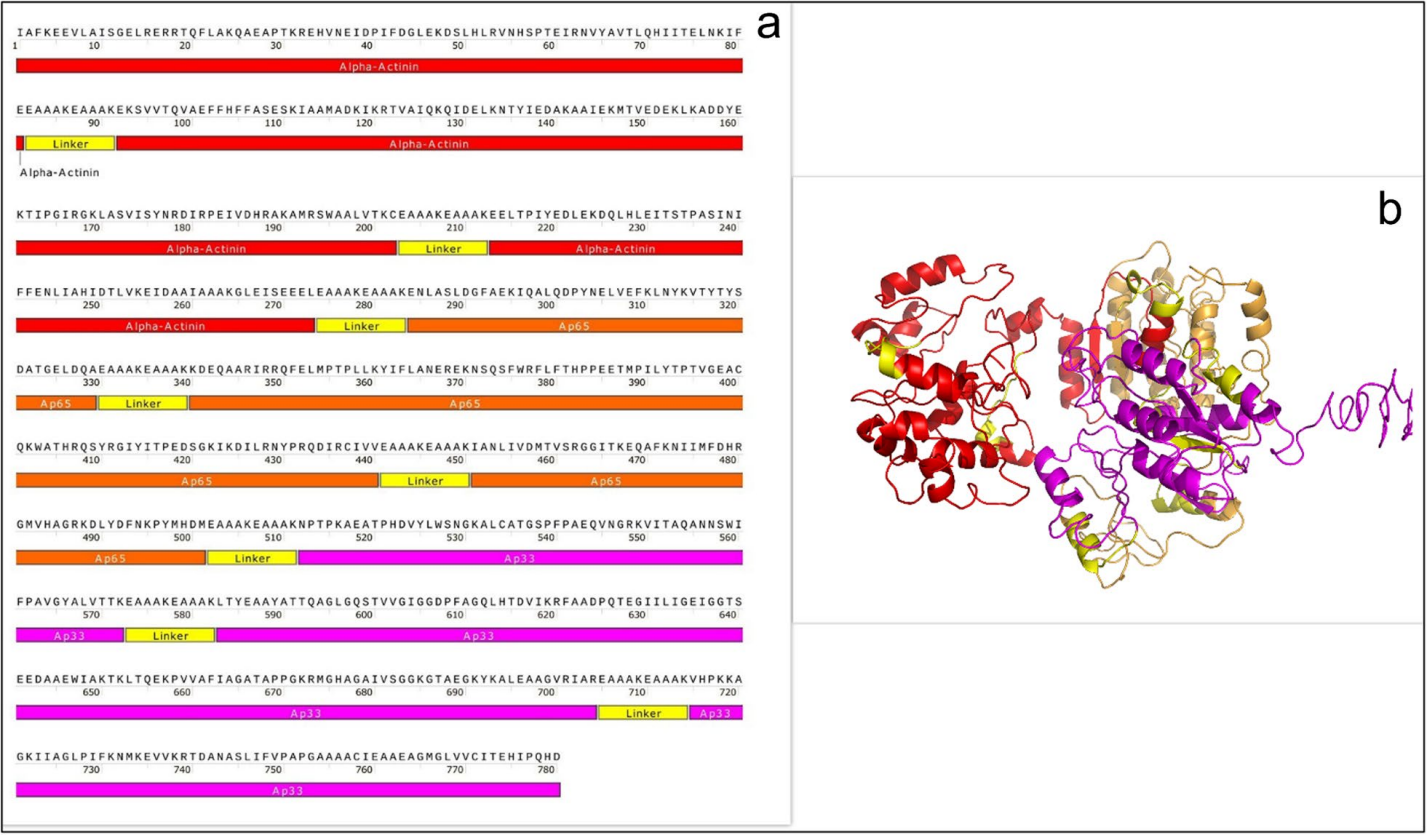
**a** Schematic diagram of the final construct of the multiepitope protein. **b** The tertiary structure of the designed protein

By combining selected epitope-rich domains at different positions using EAAAK, EAAAKEAAAK, and GGGGS linkers, several protein constructs were designed. The designed constructs were evaluated based on physicochemical properties, antigenicity, and secondary and tertiary structure, and finally the most suitable construct was introduced as a vaccine candidate (Additional file 3: S3) (Fig. 1a, b).

### Physical and chemical properties of designed structure

Using the EXPASY ProtParam server (http://expasy.org/tools/protparam.html), the physical and chemical properties of the designed structures such as the number of amino acids, molecular weight, PI, number of charged amino acids, amino acid composition, hydrophobicity, and hydrophilicity were obtained. The results of this investigation showed that our designed vaccine candidate protein finally consisted of 780 amino acids and had a molecular weight of 85,190.25 daltons (Table 2). The instability index (< 40) indicates that the designed protein has high stability to induce an immunogenic response. The instability index of our vaccine candidate was 35.8, which classifies the protein as stable. The aliphatic index of the recombinant protein was calculated to be 86.04, indicating the stability of this protein at different temperatures (Table 2).

**Table 2:** Evaluation of physical and chemical properties of the designed structure using the EXPASY ProtParam Server

The physiochemical properties	Analyze
Number of amino acids	780
Molecular weight	85,190.25
Theoretical PI	5.98
Total number of negatively charged residues (Asp + Glu)	112
Total number of positively charged residues (Arg + Lys)	100
Estimated half-life	20 h (mammalian reticulocytes, in vitro), 30 min (yeast, in vivo), > 10 h (*E. coli*, in vivo)
Instability index	35.80
Aliphatic index	86.04
Grand average of hydropathicity (GRAVY)	−0.278

### Antigenicity, allergenicity, and solubility evaluation

The Vaxijen 2.0 server predicts the designed protein as an antigen with a threshold score ≥ 0.4 (score: 0.4983). The Evaller web server was used to check the allergenicity of the designed structure. The designed protein was not allergenic. The solubility of the vaccine candidate was also evaluated using the Protein-sol server. Our selected protein has a solubility score of 0.555. Solubility-scaled proteins using the Protein-sol server that have a score greater than 0.45 indicate a higher solubility than the average soluble *E. coli* protein from the experimental solubility dataset [45]; therefore, our designed protein has a high solubility.

### Secondary and tertiary structure prediction and validation

**Fig. 2:**
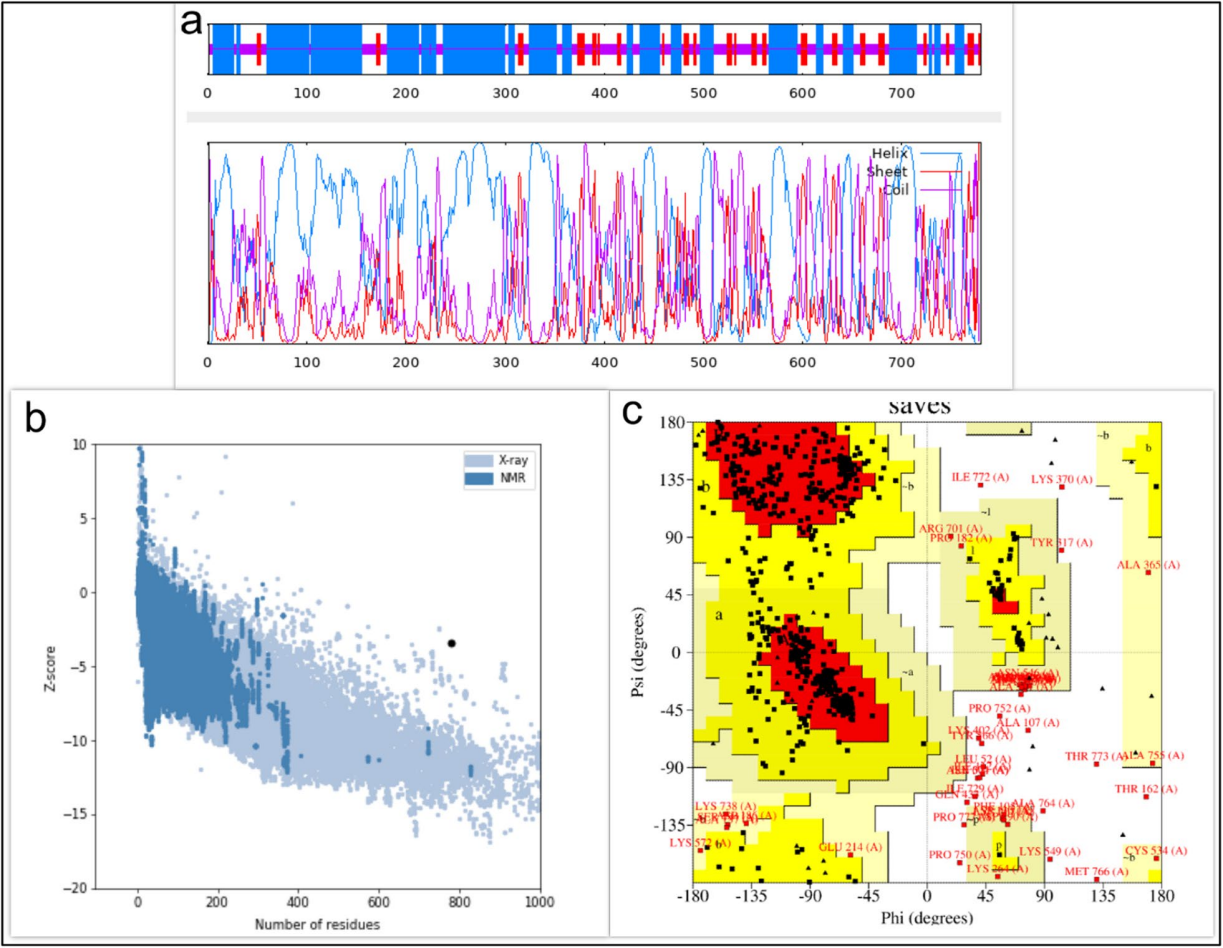
Predicting and validating the secondary and tertiary structure of the vaccine candidate. **a** Secondary structure of the designed protein. **b** Validation of the tertiary structure of the protein by ProSA-web. **c** Validation of the tertiary structure of the protein by Ramachandran plot

The GOR software was used to check the second structure of the designed structures. The amino acids that make up these recombinant proteins are involved in the formation of random coils, alpha helixes, and beta strands. The results showed that out of 780 amino acids, 430 amino acids (55.13%) are alpha helix, 96 amino acids (12.31%) are extended strands, and 254 amino acids (32.56%) are random coils (Fig. 2a). Tertiary structures were predicted by the I-TASSER server for designed protein sequences. All structures were validated and the best structure was selected. Predicted tertiary structures were evaluated using the MolProbity, ProSA-web, and SAVES servers. The MolProbity server was used to evaluate the structural similarity of new proteins to the best-known structures of similar proteins (http://molprobity.biochem.duke.edu/help/validation_options/summary_table_guide.html). On MolProbity analysis, the protein structure analysis was evaluated based on the Clash score and the MolProbity score. The SAVES server (https://saves.mbi.ucla.edu/) was also used to check the Ramachandran plot and evaluate the placement of amino acids in the favored, allowed, and disallowed regions.

On MolProbity evaluation, it was found that the Clash score for this protein was 2.49 (99% similar to the structures). Also, the MolProbity score was 2.13 (69% similar to the best structures). ProSA-web analyzed a 3D model of the vaccine candidate using an energy plot and *Z*-score. ProSA-web analyzed a 3D model of the vaccine candidate using energy plot and *Z*-score. The *Z*-score of the selected protein was −3.44, which was within the range of native protein structure (Fig. 2b). The evaluation of the Ramachandran diagram also showed that 97.4% of the amino acids were in the favored and allowed region and 2.6% were in the nonallowed areas, indicating the appropriate structure predicted for the protein (Fig. 2c).

### Prediction of conformational B cell epitopes

**Fig. 3:**
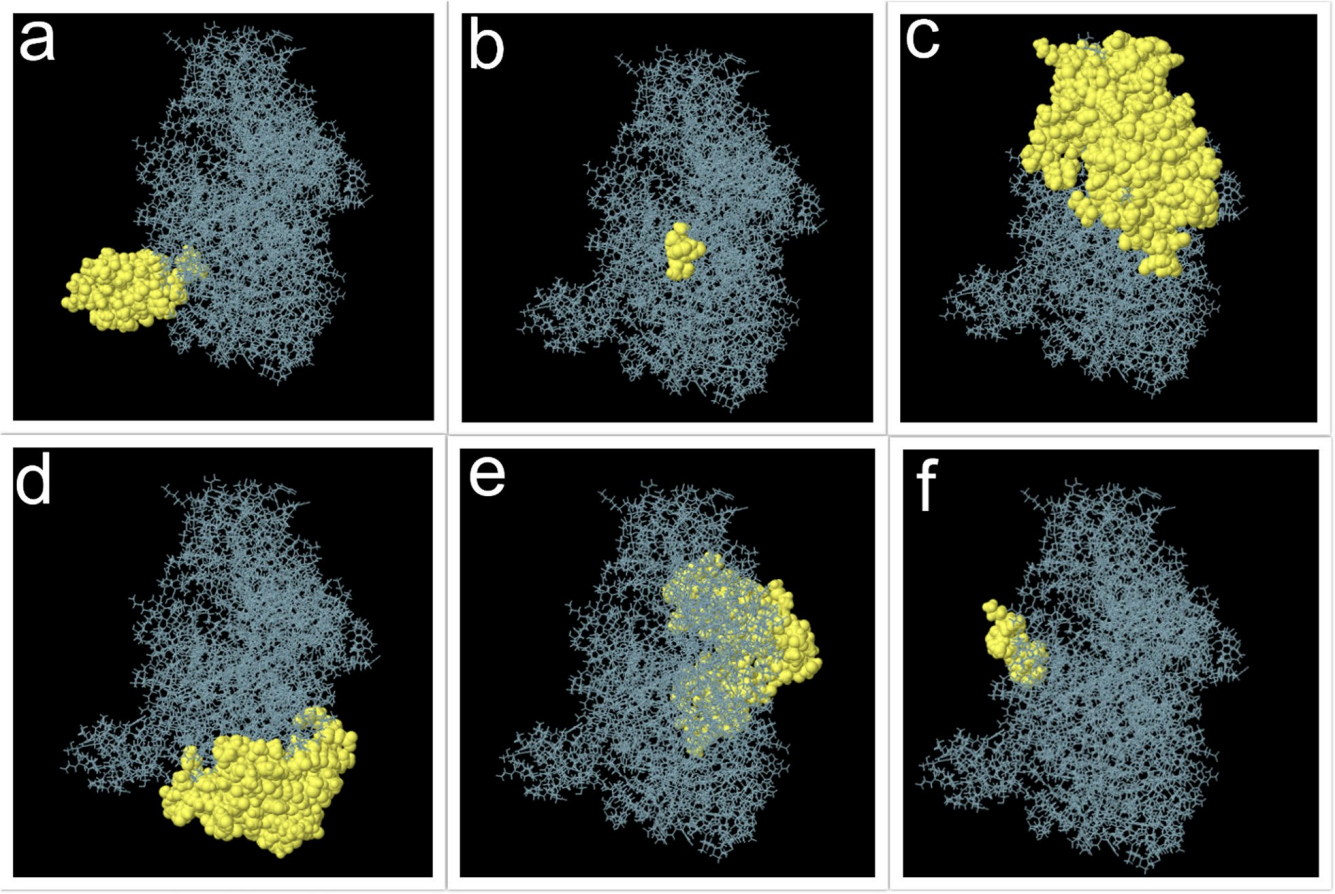
The most potent vaccine candidate conformational epitopes designed using the Ellipro server. **a** Epitope with score 0.901, **b** Epitope with score 0.736, **c** Epitope with score 0.723, **d** Epitope with score 0.657, **e** Epitope with score 0.641, **f**: Epitope with score 0.596

Ellipro servers were used to predict this type of epitope (Fig. 3a–f). The 3D structure of the designed vaccine protein used in the Ellipro server was predicted by the I-TASSER server. The most antigenic epitopes with a score above 0.5 is presented in Table 3.

**Table 3:** Prediction of B cell conformational epitopes by Ellipro

No	Residues	Number of residues	Score
1	A725, G726, L727, P728, I729, F730, K731, N732, M733, K734, E735, V736, V737, K738, R739, T740, D741, A742, N743, A744, S745, L746, I747, F748, V749, P750, A751, P752, G753, A754, A755, A756, A757, C758, I759, E760, A761, A762, E763, A764, G765, M766, G767, L768, V769, V770, C771, I772, T773, E774, H775, I776, P777, Q778, H779, D780	56	0.901
2	H71, I72, I73	3	0.736
3	G12, E13, L14, R15, R17, R18, T19, Q20, F21, L22, A23, K24, Q25, A26, E27, A28, P29, T30, K31, R32, E33, H34, V35, N36, E37, I38, D39, P40, I41, F42, D43, G44, L45, E46, K47, D48, S49, L50, H51, L52, R53, V54, N55, H56, S57, P58, T59, E60, I61, R62, V64, Y65, A66, V67, T68, L69, Q70, E75, L76, N77, K78, F80, E81, E82, A83, A84, A85, K86, E87, A88, A89, A90, K91, E92, K93, S94, V95, V96, T97, Q98, V99, A100, E101, F102, F105, F106, S110, K111, I112, A113, A114, M115, A116, D117, K118, I119, R121, V123, A124, K127, Q128, I129, D130, E131, L132, K133, N134, T135, Y136, I137, E138, D139, A140, K141, A142, A143, I144, E145, K146, M147, E150, D151, E152, K153, L154, K155, A156, D157, D158, Y159, E160, K161, T162, I163, P164, G165, I166, I180, R181, P182, E183, I184, V185, D186, H187, R188, A189, K190, A191, M192, D221, L222, E223, D225, Q226, H228, L229	157	0.723
4	E255, I256, D257, A258, A259, I260, A276, A282, K283, F292, A293, E294, Q297, D301, N304, E305, L306, V307, E308, F309, K310, L311, N312, Y313, K314, V315, T316, Y317, T318, Y319, S320, D321, A322, T323, G324, E325, L326, D341, E342, A344, A345, R346, R348, R349, Q350, F351, E352, L353, M354, P355, T356, P357, L358, L359, K360, Y361, I362, F363, L364, A365, N366, S372, S374, F375, F378, L379, F380, T381, H382, P383, P384, E385, E386, T387, M388, P389, I390, L391, Y392, T393, P394, T395, V396, Q408, Y410, R411, G412, I413, Y414, T416, P417, E418, D419, S420, G421, K422, I423, K424, D425, N429	100	0.657
5	V440, E441, A443, A444, K445, E446, A447, A448, A449, K450, A452, N453, L454, I455, D457, M458, T459, V460, S461, R462, G463, G464, I465, T466, K467, E468, Q469, A470, F471, K472, N473, I474, M476, F477, D478, H479, R480, G481, M482, V483, H484, A485, G486, R487, K488, D489, L490, Y491, F493, N494, P496, Y497, M498, H499, D500, M501, A503, A504, A505, K506, E507, A508, A509, A510, K511, P513, G530, A532, L533, C534, A535, G537, S538, P539, F540, P541, Q554, A555, N556, N557, G565, Y566, A567, L568, V569	85	0.641
6	A649, K650, T651, K652, T654, Q655, E656, K657	8	0.596

### Protein–protein molecular docking

**Fig. 4:**
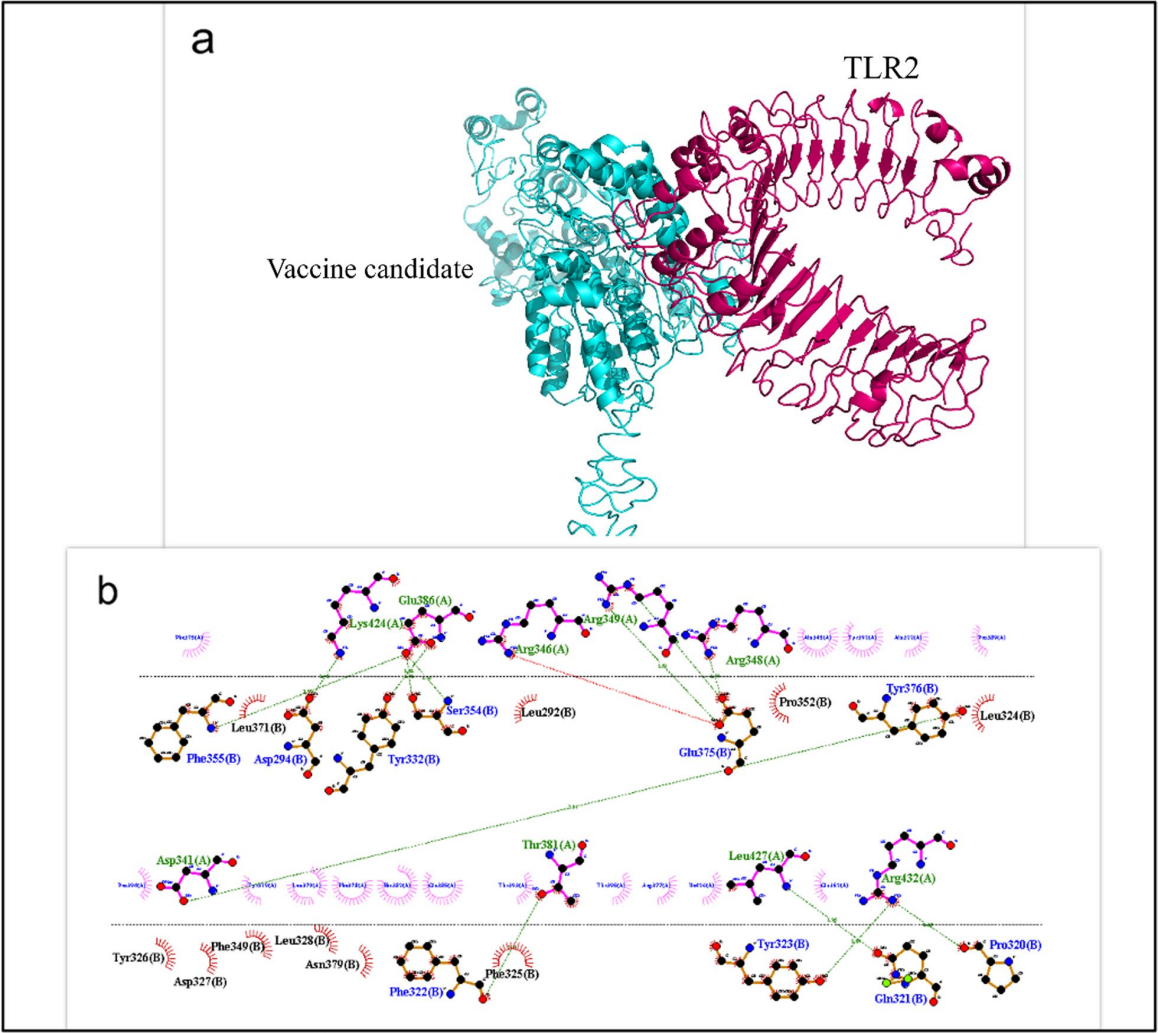
**a** Graphic representation of the interaction of the designed vaccine candidate with the TLR2 complex. **b** LIGPLOT representation of the amino acids involved in the interaction between the protein vaccine candidate and TLR2. *Hydrogen bonds between receptors (blue) and the protein vaccine candidate (green) and hydrophobic interactions with receptors (black) and the protein vaccine candidate (blue) are indicated by dark green lines

**Fig. 5:**
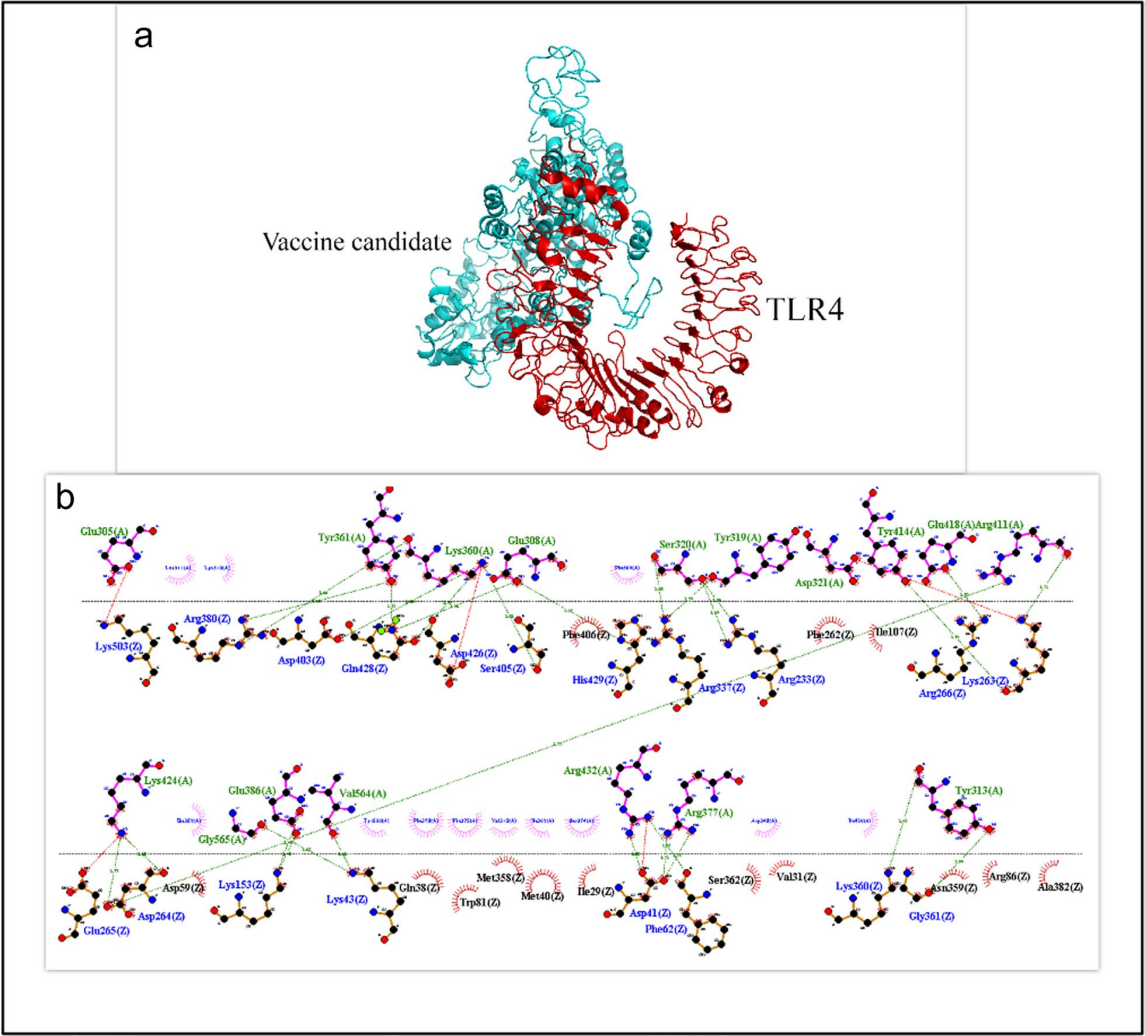
**a** Graphical representation of the interaction of the designed vaccine candidate with the TLR4 complex. **b** LIGPLOT representation of the amino acids involved in the interaction between the protein vaccine candidate and TLR4. *Hydrogen bonds between receptors (blue) and the protein vaccine candidate (green) and hydrophobic interactions with receptors (black) and the protein vaccine candidate (blue) are indicated by dark green lines

Cluspro 2.0 was used to study the protein–protein binding between the designed vaccine candidate with TLR4 and TLR2. To select the best interaction, the parameters of the weighted score and number of clusters calculated by Cluspro 2.0 were evaluated. In addition, hydrogen and hydrophobic bonds between the vaccine candidate and TLR4 and TLR2 were investigated using the LIGPLOT tool. Finally, we considered the lowest energy and the lowest affinity (Kd) obtained from the PRODIGY web server as essential standards for selecting the strongest complexes. The results showed that there is a strong interaction between the vaccine candidate with TLR4 and TLR2 (Table 4). Interactions between TLR2 (Fig. 4) and TLR4 (Fig. 5) and the designed vaccine candidate were observed using PyMOL and LIGPLOT. As shown in Figs. 4 and 5, the vaccine candidate made a strong interaction with the active site of the receptors, and this binding includes the essential amino acids Ile319, Phe322, Phe325, Tyr326, Val348, Phe349, and Pro352 for TLR2 and the amino acids Arg434, Arg380, Lys341, Lys263, and Gln339 for TLR4.

**Table 4:** Evaluation of molecular binding results between protein vaccine candidate and TLR4 and TLR2

Complex	ΔG (kcal mol^−1^)	Weighted score	Number of hydrogen bonds	Number of hydrophobic bonds	PRODIGY (Kd)
Vaccine candidate—TLR4	−14.1	−967.3	28	12	4.3e−11
Vaccine candidate—TLR2	−11.4	−890.7	13	16	4.4e−09

### Molecular dynamics simulation

**Fig. 6:**
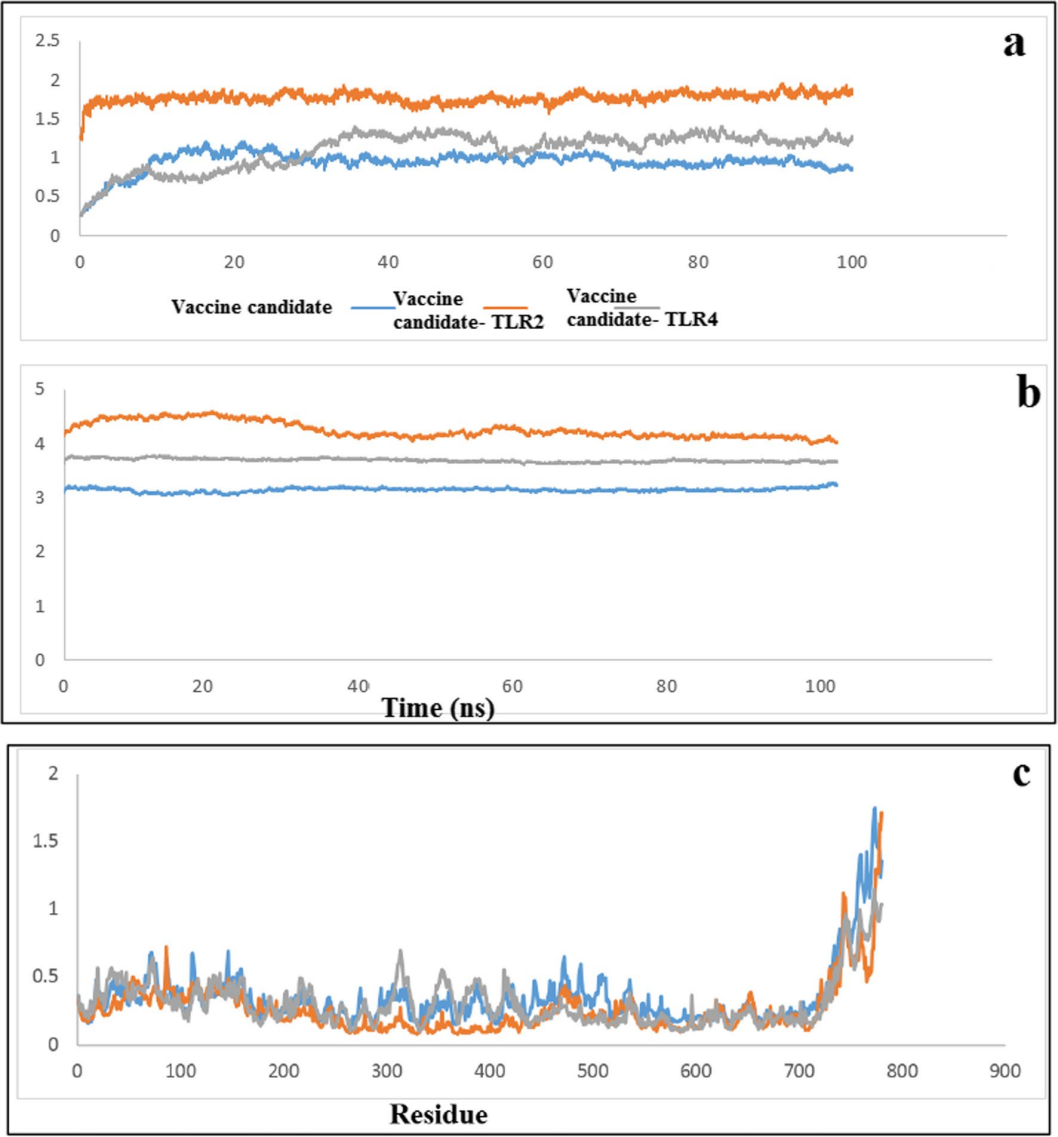
**a** RMSD results of the designed protein and protein–TLR2 and protein–TLR4 complexes in unit time (ns). **b** Rg results of the designed protein and protein–TLR2 and protein–TLR4 complexes per unit time. **c** RMSF results of the designed protein in the noninteracting form and in the interacting form with TLR2 and TLR4

To verify the stability of the designed protein structure and protein–receptor complexes, MD simulation was performed for up to 100 ns. The RMSD parameter is used when analyzing the results of MD simulations of proteins and complexes to obtain the degree of movement of the protein or atoms when the ligand is placed in the active site of the receptor and to evaluate the stability of the structure, deviation, and conformations of the protein or complex during the simulation period. A lower RMSD value indicates more stability and less fluctuations during the simulation. The analysis of the results related to the RMSD of the designed protein and the complexes showed that the designed protein reached stability after about 10 ns and its average RMSD was 0.95 nm (Fig. 6a). This stability is maintained during the simulation up to 100 ns. Also, protein–TLR2 complexes with an average of 1.7 nm are stable during the simulation (Fig. 6a). The protein–TLR4 complex reached stability after about 40 ns with an average RMSD of 1.1 nm, and considering that the fluctuations during 40–100 ns are less than 0.3 nm, it can be concluded that the complex has reached stability (Fig. 6a). Another parameter that has been investigated in the evaluation of MD simulations is the Rg, which is evaluated the amount of compression changes during the MD simulation. Rg is defined as the distribution of a protein’s atoms around its axis and is widely used in the calculation of protein behavior. Therefore, this variable allows us to analyze the overall dimensions of the protein, and the more stable the compression of the protein is during the simulation, it indicates the stability of the protein and the complexes. As the graph shows, the fluctuations of the designed protein alone and in interaction with TLR4 and TLR2 are stable during the simulation (Fig. 6b).

The RMSF of the amino acid residues can be used to evaluate the motion and flexibility of the structure. In addition, we decided to perform an RMSF analysis to examine the changes in the backbone atoms of the designed protein and the protein–TLR4 and protein–TLR2 complexes. In this analysis, the average value of changes of each residue during the simulation was plotted. As shown in Fig. 6c, the RMSF values show small fluctuations (less than 0.3 nm) for most amino acids in protein–TLR4 and protein–TLR2 complexes compared with the designed protein. These results show that the designed protein becomes more stable in interaction with the immune system receptors.

**Fig. 7:**
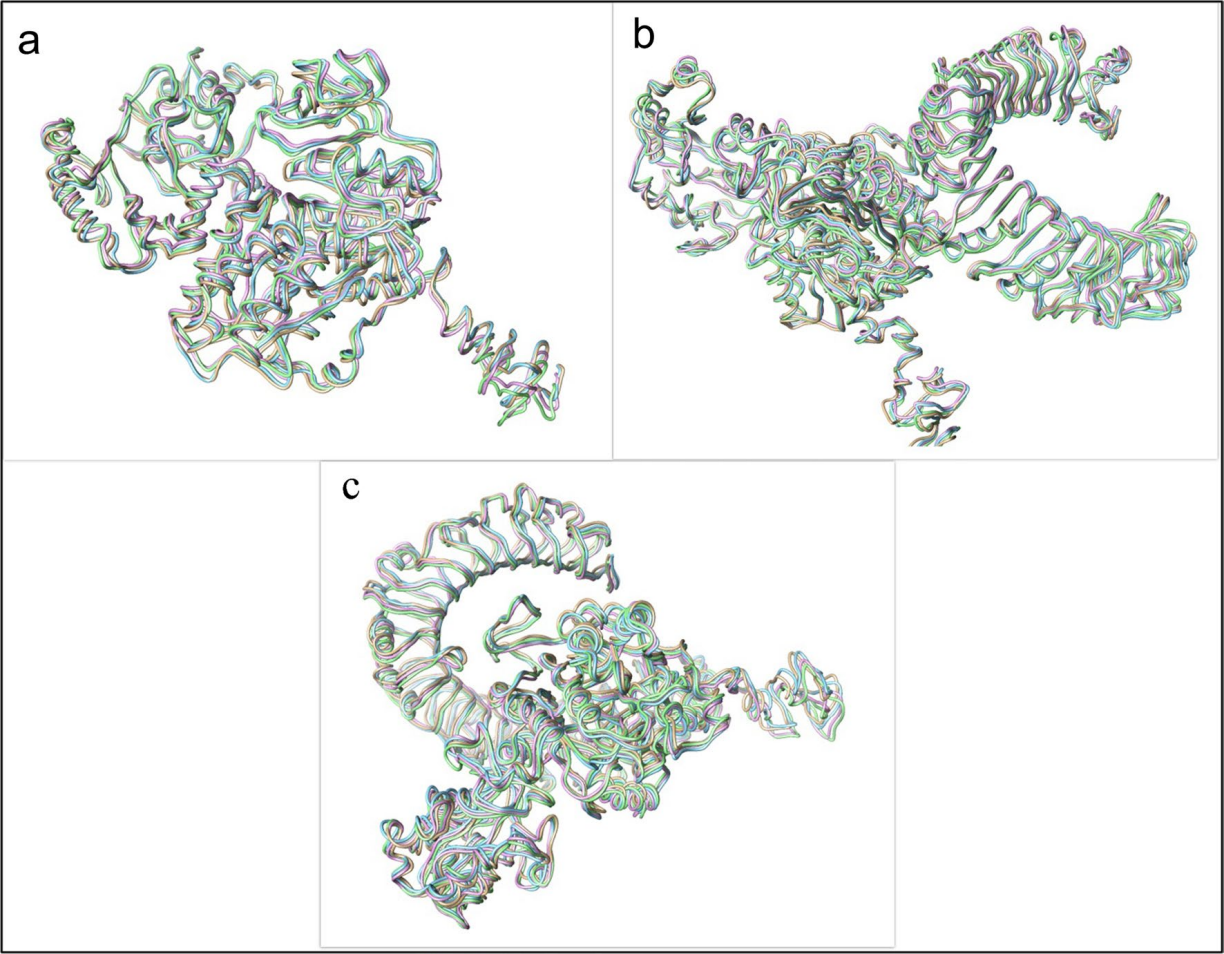
Snapshots of 0, 50, 75, and 100 ns of MD simulation of the vaccine candidate and ligand–receptor complexes. **a** The vaccine candidate, **b** vaccine candidate–TLR2, and **c** vaccine candidate–TLR4 complexes. Brown: 0 ns; blue: 50 ns; purple: 75 ns; light green: 100 ns

Snapshots taken at 0, 50, 75, and 100 ns intervals to check the state of the vaccine during the simulation showed that the structure of the vaccine and the site of interaction of the vaccine with the receptors were stable during the simulation (Fig. 7a–c).

**Fig. 8:**
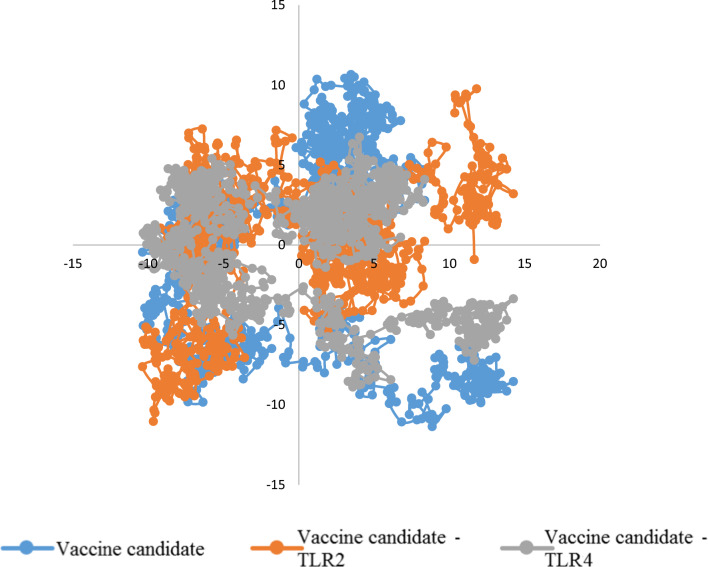
Conformational sampling in principal component analysis. Two-dimensional projection of trajectories showing conformational sampling of the vaccine candidate and vaccine candidate–TLR2 and vaccine candidate–TLR4 complexes

**Fig. 9:**
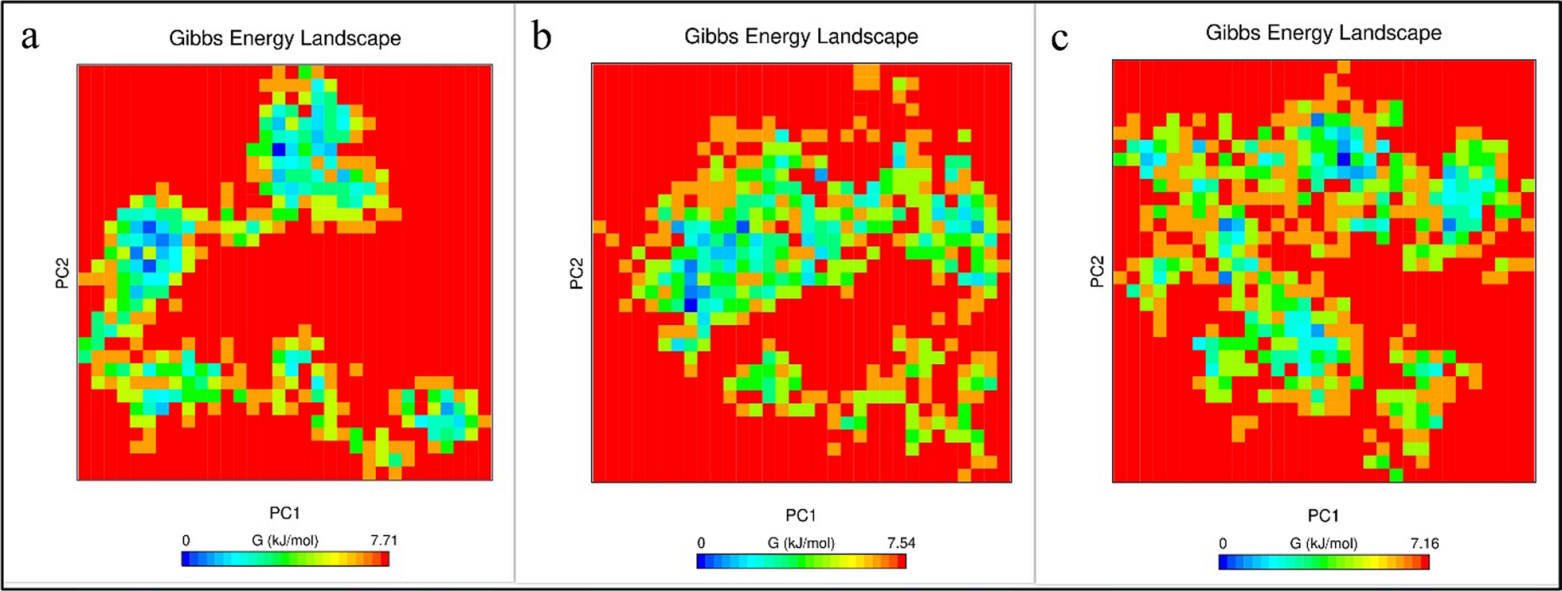
The Gibbs energy landscape plot during 100 ns of simulation. **a** The vaccine candidate, **b** Vaccine candidate-TLR2, **c** Vaccine candidate-TLR4 complexes

Using covariance matrices of Cα atoms, PCA calculates the significant motions of atom pairs associated with vital biological functions. The first two principal components (PC1 and PC2) of the candidate vaccine, candidate vaccine–TLR2 and candidate vaccine–TLR4 complexes were generated by projecting the trajectories onto their respective eigenvectors. Figure 8 shows the PCA of the three structures. The plot shows that most of the common essential subspace was occupied by the vaccine candidate–TLR2 and vaccine candidate–TLR4 complexes. In the Eigenvector (EV) plots, the three structures shared a common conformational subspace. The sampling of both systems demonstrates the stability of the complexes and the vaccine candidate in the simulation. In addition, the FELs of the first and second PCA showed that the vaccine candidate, vaccine candidate–TLR2 and vaccine candidate–TLR4 complexes had global energy minima of 7.71, 7.54, and 7.15 kJ mol^−1^, respectively (Fig. 9). The Gibbs energy landscape shows the same energy range for all three structures and it can be argued that the structures have not undergone sudden drastic changes and are stable. These results are consistent with the analysis of RMSD, Rg, and RMSF values.

### Immune simulation

**Fig. 10:**
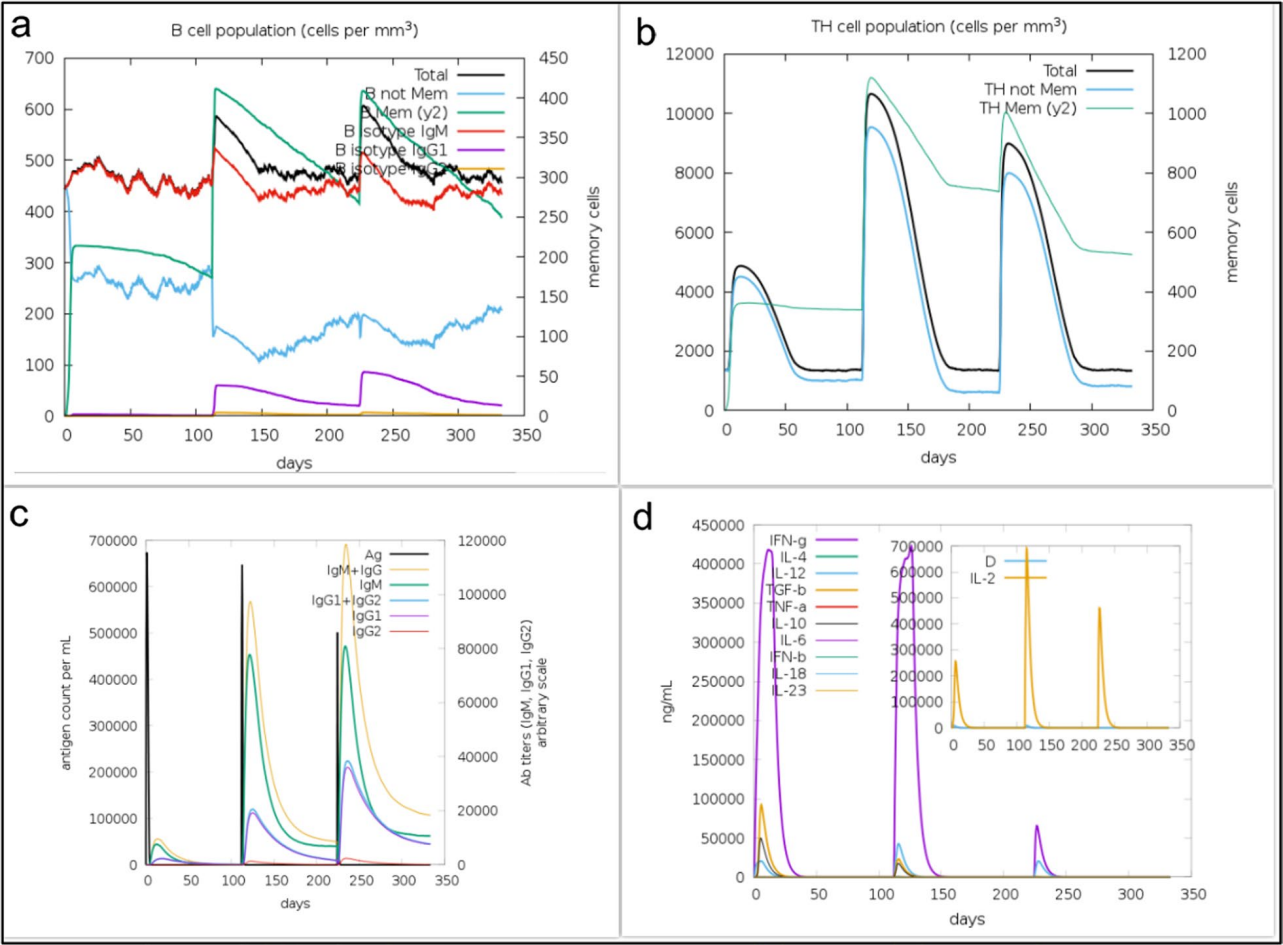
In silico immunity simulation against protein antigen designed as a vaccine candidate using C-ImmSim web server. Simulations after three injections at steps 1, 336, and 672 are presented. **a** B cell population. **b** TH cell population. **c** Antigen and immunoglobulin. **d** Cytokine production

The C-ImmSim server was used to simulate the immune system response to the designed vaccine candidate. Figure 7 shows the simulation of the host immune response to the vaccine candidate protein. Antigen and immunoglobulin parameters, cytokine production, TH cell population and B cell population were examined in this evaluation. An increase in IgM levels indicates the initial host response. In addition, a secondary response to the designed protein as antigen is indicated by increased levels of B cell population (Fig. 10a), TH cell population (Fig. 10b), and IgG1 and IgG2 (Fig. 10c). There was also a significant increase in the levels of cytokines and interleukins after immunization, especially interferon-γ (Fig. 10d). Interpretation of the results indicates that the vaccine candidate is capable of stimulating the immune system to produce cytokines and antibodies against *T. vaginalis.*

### Codon optimization and in silico cloning of the designed candidate vaccine

**Fig. 11:**
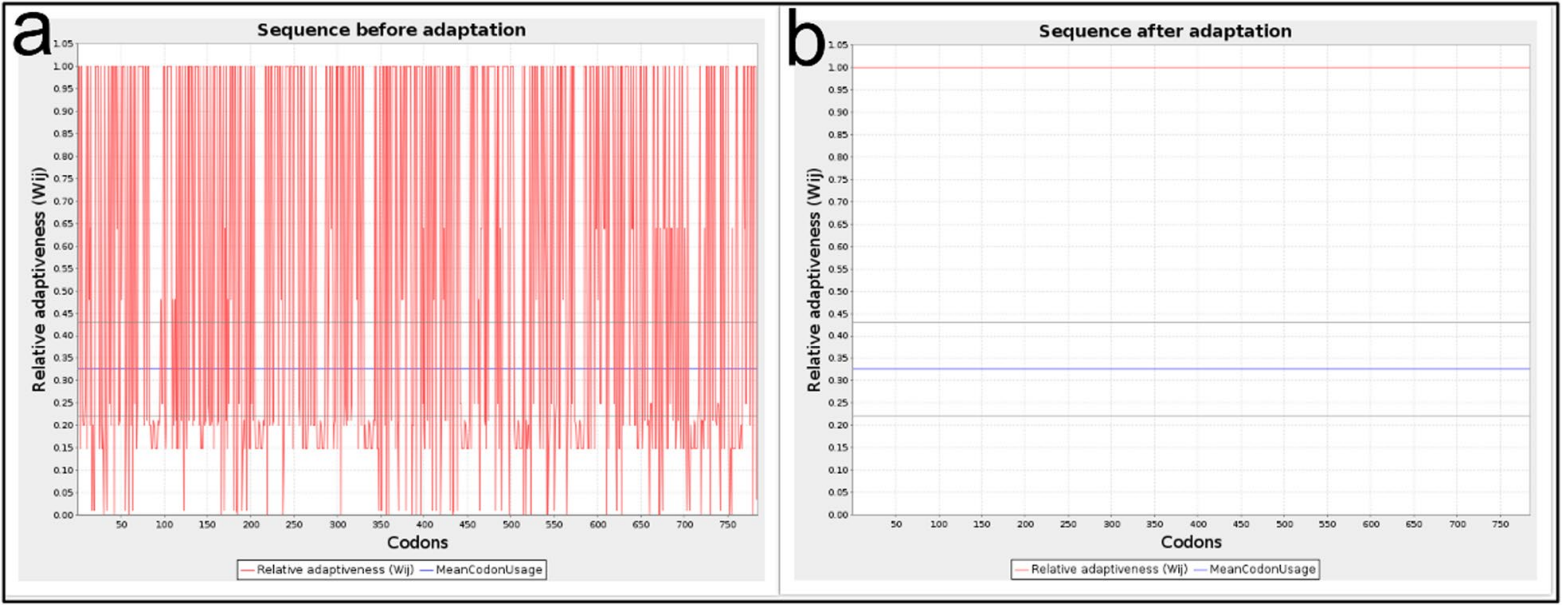
Codon optimization using the JCat web server. **a** Before optimization, **b** after optimization

**Fig. 12:**
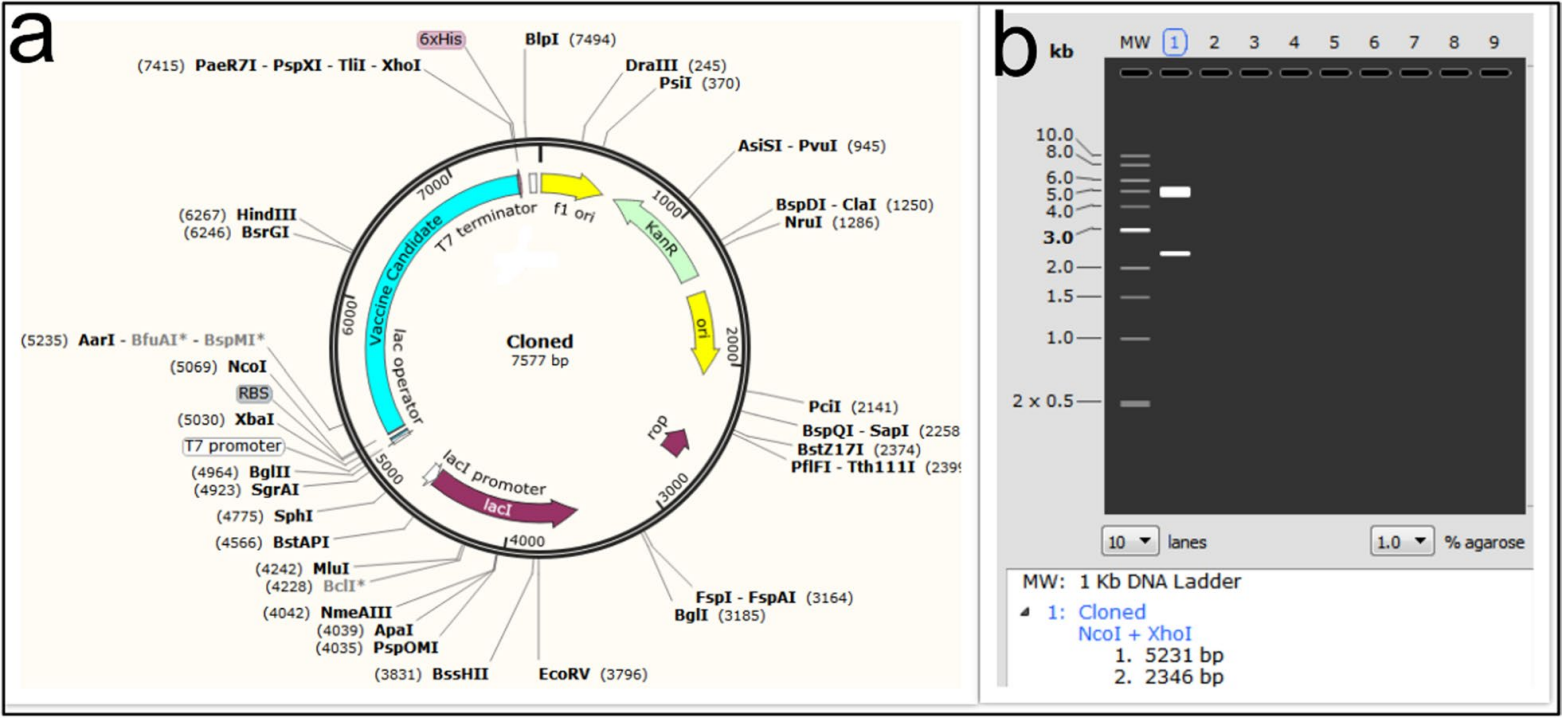
**a** Cloning of the designed protein construct into the pET-28a vector (shown in blue). **b** Informatics evaluation of the cloning of the designed protein by double digest

Codon optimization was performed using the JCat tool. After codon optimization, the sequence length of the designed structure was 2352 nucleotides. The codon compatibility index and the GC content of the nucleotide sequence before the optimization were 0.311% and 66.24%, respectively. After codon optimization, the parameters were 1% and 50.73%, respectively (Fig. 11a, b). The simulation of the optimized sequence of the vaccine candidate in pET-28a^(+)^ using the SnapGene software showed that the vaccine candidate sequence is clonable in pET-28a^(+)^ (Fig. 12a). In the middle of the designed construct, there is a cleavage site for HindIII and BsrGI enzymes, so we set the first and last sequence of the construct with NcoI and XhoI enzymes, respectively. Double digestion with NcoI and XhoI enzymes showed presence of vaccine candidate (2346 bp) together with pET-28a^(+)^ vector (5231 bp) (Fig. 12b).

## Discussion

The 5-nitroimidazole class of drugs is used almost exclusively to treat trichomoniasis. In the USA, metronidazole or tinidazole are used to treat trichomoniasis. However, because of the similarity in chemical structure, infections that are highly resistant to metronidazole may not be cured even after a standard course of treatment with tinidazole [46, 47]. Infections that are not treated with standard therapeutic doses can often be cured by prolonged treatment with the same drugs. Clearly, this is not an ideal strategy for treating drug-susceptible infections as it can lead to the selection of much more resistant strains [46]. Also, self-medication is an important issue, especially in developing countries, which leads to the increase of antibiotic resistance [48]. Increasing resistance of isolates to approved drugs and clinical complications, including increased risk of HIV infection and transmission, cervical and prostate cancer, and adverse pregnancy outcomes, may be reasons for greater attention to the development of a vaccine against this infection [1]. The development of a vaccine against *T. vaginalis* can reduce the human costs of pregnancy complications and infertility, as well as medical and social costs [49].

The design and experimental production of multisubunit polypeptide vaccines have made significant progress in recent years [13, 50, 51]. However, to date, no vaccine has been licensed to provide complete protective immunity against the parasite *T. vaginalis*.

In this study, three proteins, AP65, AP33, and α-actinin, were selected for the design of a multiepitope vaccine. Adhesion of *T. vaginalis* to vaginal epithelial cells (VECs) is complex. Surface proteins (AP65, AP51, AP33, and AP23) appear to interact with host cells through ligand–receptor type interactions [9]. The AP65 protein was identified as part of researchers’ efforts to determine which factors play a key role in adhesion. Moreover, AP65/BNIP3 interaction causes *T. vaginalis* to adhere to host cells and become pathogenic, and this protein is introduced as a basis for preventing and treating trichomoniasis [9]. The α-actinin protein is used to diagnose trichomoniasis and is an abundant immunogen in the serum of patients with this infection. This protein acts as an adhesive to host cells and is one of the pathogenic factors responsible for the pathogenesis of *T. vaginalis* [52].

Given the necessity of the AP33, AP65, and α-actinin proteins mentioned above, it was decided in this study to design a vaccine consisting of B and T cell epitope-rich domains that will ultimately be effective in the event of human exposure to *T. vaginalis*; this parasite is targeted by the immune system to prevent infection. In the present study, based on the score obtained by epitope prediction tools, nine epitope-rich domains were selected from these three proteins, containing B cell and T cell epitopes, and linked together by EAAAKEAAAAAK linkers. Ideally, epitope vaccines should include B cell epitopes that stimulate a protective antibody response and also essential T cell epitopes that stimulate cytotoxic T lymphocyte (CTL) production and a Th immune response. The final sequence of our vaccine candidate was an antigenic, nonallergenic protein that was soluble when overexpressed in *E. coli*. Evaluation of the tertiary structure using the MolProbity, ProSA-web, and SAVES servers indicated that it closely resembled the tertiary structures of proteins found in nature and that most amino acid residues (97.4%) were in the preferred region. In addition, the vaccine candidate was analyzed for its interaction with TLR2 and TLR4 of the immune system, which play an important role in fighting *T. vaginalis* infection, after confirming the physicochemical properties and evaluating the tertiary structure of the designed protein. Pattern recognition receptors, particularly TOL-like receptors, are one of the major immune strategies used by immune cells to recognize *T. vaginalis*, especially TLR2 and TLR4. This suggests a possible immune mechanism in epithelial cells during parasite infection [18]. The result of molecular docking using the Cluspro and PRODIGY servers showed that this recombinant protein efficiently binds to the active site of TLR2 and TLR4 and can induce an immune response. The designed protein binds strongly to both TLR4 [ΔG: −14.1 (kcal mol^−1^)] and TLR2 [ΔG: −11.4 (kcal mol^−1^)]. However, the interaction with TLR4 is stronger. To evaluate the stability of the designed protein structure as well as the interaction stability of protein–TLR2 and protein–TLR4 complexes, MD simulation was performed up to 100 ns. The analysis of the RMSD results of the designed protein and the complexes showed that the designed protein and the TLR2–protein complex were quite stable during the simulation. The protein–TLR4 complex had fluctuations of less than 0.3 nm after 40 ns, indicating the stability of the complex. The Rg parameter, which is analyzed to check the amount of compression changes during the MD simulation, allows us to analyze the overall dimensions of the protein, and the more stable the protein compression is during the simulation indicates the stability of the receptor–ligand interaction. The compression of all three structures was stable in this study. The degree of movement and flexibility of the residues of the structure is measured using RMSF. Our evaluations showed that the amino acids in the unbound protein structure and the complexes were stable during the simulation with fluctuations of less than 0.3 nm. By analyzing snapshots from different times of the molecular dynamics simulation, we ensured that the binding site of the vaccine candidate to the receptor was stable during the simulation and that no conformational changes occurred. Another check we performed to confirm the simulation analyses was PCA calculations. In these calculations, by analyzing the EV plots and also evaluating the Gibbs global energy, we concluded that the structure of the unbound protein as well as the complexes had not undergone drastic changes and were stable. It can be argued that the vaccine candidate protein interacts strongly and is stable with the receptors of the immune system, which can lead to initiate the production of the innate immune response and ultimately acquired immunity [53]. *T. vaginalis*-specific antibodies and T cell-mediated immune responses are effective in eliminating the parasite [16]. Studies conducted in the development and production of vaccines against *T. vaginalis* show that the most important cytokines and antibodies to eliminate this parasite are specific total IgG and subtypes (IgG1 and IgG2a) and the cytokines IL-4, IL-10, IFNγ, and IL-6 [2, 8]. The simulation of the immune response generated in the body using the C-ImmSim server showed that the designed vaccine candidate can generate a necessary immune response by increasing the level of antibodies and cytokines necessary to fight *T. vaginalis.* The results of the present study may be a guide for future experimental studies for a better understanding of the biological functions of the designed protein as a vaccine candidate. The most popular expression plasmid on the market is pET28a. Therefore, we cloned the designed construct into this plasmid. The analysis of this evaluation showed that the designed construct can be cloned into pET28a with cleavage sites of NcoI and XhoI enzymes at the first and last sequence, respectively.

## Conclusions

We have analyzed the three *T. vaginalis* proteins, AP33, AP65, and α-actinin, for the best immunogenic domains for the induction of a robust immune response. Finally, a protein vaccine candidate against *T. vaginalis* was designed by selecting nine domains rich in B and T cell epitopes and linking them to the EAAAKEAAAK linker. The sequence selected as a vaccine candidate creates a protein structure that is stable and capable of interacting with the TLR2 and TLR4 receptors of the immune system as an immunogen and elicits the appropriate potential response to provide effective protection.

### Supplementary Information

**Additional file 1: Table S1.** Selected Linear B cell Epitopes from *Trichomonas vaginalis*.

**Additional file 2: Table S2.** Selected High-Affinity Binding human MHC Class II Epitopes from *Trichomonas vaginalis*.

**Additional file 3: S3.** The final vaccine sequence.

## Data Availability

The data supporting the conclusions of the study are available within the article and/or its supplementary materials.

## Abbreviations

*T. vaginalis*: *Trichomonas vaginalis*
IEDB: Immune Epitopes and Analysis Resource
ns: Nanoseconds
RMSF: Root mean square fluctuations
RMSD: Root mean square distance
RG: Radius of gyration
