# Supplementary material for: Introduction of protein vaccine candidate based on AP65, AP33, and α-actinin proteins against Trichomonas vaginalis parasite: an immunoinformatics design
Source: Parasit Vectors. 2024 Mar 31;17:165. doi: 10.1186/s13071-024-06248-y (PMC10981826; doi:10.1186/s13071-024-06248-y)
Supplement: Supplementary file 1 — Additional file 1: Table S1. Selected Linear B cell Epitopes from Trichomonas vaginalis. [file 13071_2024_6248_MOESM1_ESM.docx]

**Table S1. Selected Linear B cell Epitopes from *Trichomonas vaginalis***

| Server | antigen | Start position | End position | peptide |
| --- | --- | --- | --- | --- |
| Bepipred | **Ap33** | 5  16  55  63  103  117  135 | 7  44  60  97  108  122  202 | GLS  GAGSLLPCDGVTKKKNNVIKVKFTEEEDL  AKDWIR  QLMITRNPRQCRERWNNYINPALRTDPWSPEEDML  AEYGPK  KNRSDN  RAKHQKSPISPPPTQRVEYTTPVVMPVVKEIRVAETRPLENPFESLTVQESSFSVDTDYNQFDLWND |
|  | **Ap65** | 5  72  76  89  124  141  150  152  208  244  258  300  332  357  396  423  449  502  537  556 | 64  73  76  90  130  141  150  152  234  245  262  303  339  374  398  435  460  506  548  564 | SVSVPVRNICRAKVPTLKTGMTLLQDGDLSKGSAFTKEERDRLNLRLLPYKVFTKDEQA  EL  T  EK  ATHRQSY  G  Y  R  DRKEILADPLYHGWRHPRIRGPEHTKF  EV  EMETA  VPDL  GGITKEQA  KDLYDFNKPYMHDMEVYG  GLI  PKAEATPHDVYLW  PAEQVNGRKVIT  KEDHD  LATAVPPKGTSL  LFEPSADYE |
|  | **α-actinin** | 5  34  59  92  98  125  149  151  175  207  232  332  391  592  596  598  615  790  792  832 | 17  35  75  95  107  134  149  161  190  225  328  387  558  592  596  613  787  790  824  844 | GREGLLDDAWEKT  QI  KEPMPGKWHKQPKMMVQ  KIRT  IGADDIINKN  EEISVEEATA  Y  HVAVNNFTTSW  RPNLLDYSALDYNDHK  VYLDPEDVIDTTPDEKSVV  FHFFASESKIAAMADKIKRTVAIQKQIDELKNTYIEDAKAAIEKMTVEDEKLKADDYEKTIPGIRGKLAS VISYNRDIRPEIVDHRAKAMRSWAA  KSGNRPIPEIPQGLEPEALTNKFNEIEQTSTTRRDELTQELNDMIKKKVEDFMAKC  INKCDAIHEEVKTIEGTTAEKKDKVEQKLHEAEDLQPALAELTPLFQELVELRINTLSSQTDDSVNRHHSQLITYIKHLLEQLNGKLFEETNEARINEYNALAQPLYDEAIAFKEEVLAISGELRERRTQFLAKQAEAPTKREHVN  V  D  ITALVTSSHQIPGDAA  VKAQVEENLASLDCVRRKDPSPPGSIQRARSIQAQLIKVTYTYSDATGELVQARLDLKQIILAKKTFLEEEERKARINNYTVKADEHMNEAHALDGKINSVDGELEPKRQKLYEVREEVNAKKEKAVEELTPI  A  AKGLEISEEELNEFKDTFKYFDKDKSNSLEYFE  LGEDITDDQAKEY |

| peptide | End position | Start position | Antigen | Server |
| --- | --- | --- | --- | --- |
| HQPLLFI  TRVVIQG  TKVVGAVHPK  ASLIFVPAPGAAAACIEA  GLVVCITEHI  GCQLIGPNCPGLI  GIVSRS  QSTVVGI  HTDVIKR  GIILIG  EKPVVAFIAGA  AAGVRIA | 17  27  53  96  111  139  166  190  206  220  252  289 | 11  21  44  79  102  127  161  184  200  215  242  283 | **Ap33** | **IEDB (Kolaskar and Tongaonkar)** |
| EK  ATHRQSY  G  Y  R  DRKEILADPLYHGWRHPRVRGAEHLKF  EV  EMETA  VPDL  GGITKEQA  KDLYDFNKPYMHDMEVYG  GLI  PKAEATPHDVYLW  PAEQVNGRKVIT  KEDHD  LATAVPPKGTSL  LFEPSADYE | 90  130  141  150  152  234  245  262  303  339  374  398  435  460  506  548  564 | 89  124  141  150  152  208  244  258  300  332  357  396  423  449  502  537  556 | **Ap65** |  |
| QIKVFSRWVQKQLLARQ  LLNLLEI  DLALKYINEV  DALLLW  YEHVAVN  AFAALINK  PNLLDYSAL  GACEKAFAACKELGIYVYLDPE  KSVVTQVAEFFHFFA  RTVAIQK  KLASVISY  PEIVDHR  AALVTKC  MDIINKCDAIHEEV  LQPALAELTPLFQELVELR  NRHHSQLITYIKHLLEQ  ALAQPLYDEA  KEEVLAIS  DSLHLRVNH  RNVYAVTLQHIIT  ATAVPIIDGITALVTSSHQ  DAAAVKAQ  KIQALQD  NELVEFK  NYKVTYT  QARLDLKQIILAKK  LTPIYE  DQLHLEIT  ENLIAHIDTLVKEI  AAIAAA  YFELKACLTA  KEYCKKY  QNQPVLTDA  AAYLRSQ | 33  55  89  140  154  172  183  211  235  255  304  316  330  400  442  471  499  510  555  573  606  617  637  646  654  677  647  759  783  790  829  846  894  911 | 17  49  80  135  148  165  175  190  221  249  297  310  324  387  424  455  490  503  547  561  588  610  631  640  648  664  642  752  770  785  820  840  886  905 | **α-actinin** |  |
