# Supplementary material for: Introduction of protein vaccine candidate based on AP65, AP33, and α-actinin proteins against Trichomonas vaginalis parasite: an immunoinformatics design
Source: Parasit Vectors. 2024 Mar 31;17:165. doi: 10.1186/s13071-024-06248-y (PMC10981826; doi:10.1186/s13071-024-06248-y)
Supplement: Supplementary file 2 — Additional file 2: Table S2. Selected High-Affinity Binding human MHC Class II Epitopes from Trichomonas vaginalis. [file 13071_2024_6248_MOESM2_ESM.docx]

**Table S2. Selected High-Afﬁnity Binding human MHC Class II Epitopes from *Trichomonas vaginalis***

| HLADRB 1301 | HLADRB 1101 | HLADRB 0801 | HLADRB 0701 | HLADRB 0401 | HLADRB 1501 | HLADRB 0301 | HLADRB 0101 | antigen | Server |
| --- | --- | --- | --- | --- | --- | --- | --- | --- | --- |
| _ | 724-733  AWGEVKRQI  405-414  LKKEGWRPR  434-443  WAEENSRLL  406-415  KKEGWRPRR  244-253  DGWNLPGGG  664-673  MNDQLMFLE  529-538  GIASGRARY  395-404  VHEIVRSFG  494-503  YESWTKKSP  12-21  VATARRPRW  723-732  KAWGEVKRQ  55-64  KHNMKAFLD  27-36  VLAGGFFLL | 684-693  RPFYRHVIY  563-572 EKFYDPMFK  124-133 HPNYISIIN  549-558 YPLYHSVYE  730-739 RQIYVAAFT  521-530 FEVFFQRLG  556-565 YETYELVEK  609-618 DKIYSISMK  340-349 QKVKMHIHS  567-576 DPMFKYHLT | 85-94  GTEQNFQLA  653-662  FDKSNPIVL  20-29  WLCAGALVL  242-251  YPDGWNLPG  585-594  FELANSIVL  733-742  YVAAFTVQA  356-365  YNVIGTLRG  299-308  YYDAQKLLE  18-27  PRWLCAGAL | 733-742  YVAAFTVQA  356-365  YNVIGTLRG  434-443  WAEENSRLL  381-390  WVFGGIDPQ  242-251  YPDGWNLPG  213-222  KVKNAQLAG  612-621  YSISMKHPQ  22-31  CAGALVLAG  283-291  IAEAVGLPS  570-579  FKYHLTVAQ  573-582  FLYNFTQIP  160-169  PFSAFSPQG  635-644  AVKNFTEIA  537-546  YTKNWETNK  164-173  FSPQGMPEG  399-408  VRSFGTLKK  493-502  LYESWTKKS  279-288  YRRGIAEAV  20-29  WLCAGALVL  77-86  FTQIPHLAG  660-669  VLRMMNDQL  461-470  TLRVDCTPL  661-670  LRMMNDQLM  32-41  FFLLGFLFG  737-746  FTVQAAAET  333-342  FTGNFSTQK  398-407  IVRSFGTLK  735-744  AAFTVQAAA  299-308  YYDAQKLLE  724-733  AWGEVKRQI  572-581  YHLTVAQVR  85-94  GTEQNFQLA  462-471  LRVDCTPLM  687-696  YRHVIYAPS  566-575  YDPMFKYHL  186-195  FKLERDMKI  11-20  AVATARRPR  96-105  IQSQWKEFG  672-681  ERAFIDPLG  300-309  YDAQKLLEK  99-108  QWKEFGLDS  686-695  FYRHVIYAP  371-380  YVILGGHRD  90-99  FQLAKQIQS  525-534  FQRLGIASG  209-218  FRGNKVKNA  405-414  LKKEGWRPR  417-426  LFASWDAEE  427-436  GLLGSTEWA  575-564  TVAQVRGGM  118-127  SYPNKTHPN  127-136  YISIINEDG  8-17  TDSAVATAR | 396-405  HEIVRSFGT  46-55  SNEATNITP  636-645  VKNFTEIAS  563-572  EKFYDPMFK  423-432  AEEFGLLGS  523-532  VFFQRLGIA  232-241  ADYFAPGVK  399-408  VRSFGTLKK  680-689  GLPDRPFYR  380-389  SWVFGGIDP  474-483  VHNLTKELK  55-64  KHNMKAFLD  730-739  RQIYVAAFT  726-735  GEVKRQIYV  158-167  VPPFSAFSP  702-811  GESFPGIYD  252-261  GVQRGNILN  206-215  GKVFRGNKV  36-45  GFLFGWFIK  353-342  TRIYNVIGT | 564-573  KFYDPMFKY  131-140  INEDGNEIF  103-112  FGLDSVELA | 566-575  YDPMFKYHL  279-288  YRRGIAEAV  733-744  YVAAFTVQA  55-64  KHNMKAFLD  277-286  YAYRRGIAE  572-581  YHLTVAQVR  410-419  WRPRRTILF  434-443  WAEENSRLL  327-336  YNVGPGFTG  421-430  WDAEEFGLL  219-228  LAGAKGVIL  585-594  FELANSIVL  71-80  KKFLYNFTQ  20-29  WLCAGALVL  216-225  NAQLAGAKG  371-380  YVILGGHRD  77-86  FTQIPHLAG  75-84  YNFTQIPHL  127-136  YISIINEDG  90-99  FQLAKQIQS  524-533  FFQRLGIAS  687-696  YRHVIYAPS  633-642  FSAVKNFTE  386-395  IDPQSGAAV  530-539  IASGRARYT  506-515  FSGMPRISK | **Ap33** | Rankpep |
| - | 220-229  GWRHPRVRG  434-443  LWSNGKALC  217-226  LYHGWRHPR  311-320  IGAGSAAIG  218-227  YHGWRHPRV  548-557  LKDWLKAQL  89-98  EKNSQSFWR  263-272  FKLLDHFRW  433-442  YLWSNGKAL  432-441  VYLWSNGKA  121-130  QKWATHRQS  324-333  IVDMTVSRG | 68-97  RRQFELMPT  412-421  HPVIMPLSN  93-102  QSFWRFLFT  508-517  GNLLPPLNK  114-123  PTVGEACQK  267-275  DHFRWRCNC | 433-442  YLWSNGKAL  120-129  CQKWATHRQ  112-131  YTPTVGEAC  285-294  AVAAATLAS  460-469  TAQANNSWI  257-266  FEMETAFKL  461-470  AQANNSWIF | 96-105  WRFLFTHPP  285-294  AVAAATLAS  473-482  GYALVTTKA  455-464  GRKVITAQA  120-129  CQKWATHRQ  71-80  FELMPTPLL  434-443  LWSNGKALC  284-273  AAVAAATLA  67-76  IRRQFELMP  433-442  YLWSNGKAL  310-319  FIGAGSAAI  435-444  WSNGKALCA  2-11  LTSSVSLPA  95-104  FWRFLFTHP  217-226  LYHGWRHPR  417-426  PLSNPTPKA  21-30  LKTGMTLLQ  460-469  TAQANNSWI  220-229  GWRHPRVRG  155-164  IRCIVVTDA  366-375  YMHDMEVYG  531-540  YLIDNELAT  474-483  YALVTTKAR  218-227  YHGWRHPRV  478-487  TTKARHCPA  150-159  YPRQDIRCI  20-29  TLKTGMTLL  130-139  YRGIYITPE  189-198  LIGQVDPDQ  423-432  PKAEATPHD  290-299  TLASATHME  422-431  TPKAEATPH  58-67  FTKDEQAAR  401-410  EIVQATLKN  289-298  ATLASATHM  282-291  GTAAVAAAT  487-496  KVFEIAAES | 337-346  EQAFKNIIM  52-61  LLPYKVFTK  453-462  VNGRKVITA  504-513  DHDQGNLLP  55-64  YKVFTKDEQ  184-193  LMLYTLIGQ  251-260  LVQFEDFEM  46-55  RFNLRGLLP  303-312  LKNQKIIFI  65-74  ARIRRQFEL  370-379  MEVYGSVLE  391-400  VSGVPGLIT  11-20  RELSRKVLP  471-480  AVGYALVTT | 58-67  FTKDEQAAR  344-353  IMFDHRGMV  205-304  MGTDRKEIL | 71-80  FELMPTPLL  468-477  IFPAVGYAL  520-529  YSFGIAYDV  474-483  YALVTTKAR  283-292  TAAVAAATL  257-266  FEMETAFKL  280-289  IEGTAAVAA  112-121  YTPTVGEAC  311-320  IGAGSAAIG  81-90  YIFLANERE  308-317  IIFIGAGSA  285-294  AVAAATLAS  533-542  IDNELATAV  282-291  GTAAVAAAT | **Ap65** |  |
| _ | 85-94  YINEVKKIR  65-74  WHKQPKMMV  115-124  WTCINKFMI  18-27  IKVFSRWVQ  64-73  KWHKQPKMM  316-325  RAKAMRSWA  160-169  WNTGLAFAA  21-30  FSRWVQKQL  69-78  PKMMVQKRE | 743-752  TPIYEDLEK  561-570  RNVYAVTLQ  517-526  RTQFLAKQA  766-785  NIFFENLIA  547-556  DSLHLRVNH  538-547  DPIFDGLEK  193-202  EKAFAACKE  722-731  QKLYEVREE  474-483  GKLFEETNE  231-240  FHFFASESK | 133-142  ARDALLLWA  496-505  YDEAIAFKE  921-1000  FADWVNSLY  140-149  WAKKNTQGY  842-851  YCKKYNSKG  639-848  YNELVEFKL  115-124  WTCINKFMI  922-931  ADWVNSLYN | 322-331  SWAALVTKC  139-148  LWAKKNTQG  23-32  RWVQKQLLA  562-571  NVYAVTLQH  868-877  FSKAETTET  157-166  TTSWNTGLA  317-326  AKAMRSWAA  628-637  FAEKIQALQ  521-530  LAKQAEAPT  880-889  AFKAIAQNQ  319-328  AMRSWAALV  65-74  WHKQPKMMV  900-909  FSAEDAAYL  779-786  LVKEIDAAI  449-458  ELRINTLSS  323-332  WAALVTKCK  75-84  KRENVDLAL  582-591  MVANFDATA  24-33  WVQKQLLAR  127-136  SVEEATARD  21-30  FSRWVQKQL  230-239  FFHFFASES  501-510  AFKEEVLAI  820-829  YFELKACLT  883-892  AIAQNQPVL  316-325  RAKAMRSWA  20-29  VFSRWVQKQ  679-688  FLEEEERKA  860-869  YVRFMLDHF  907-916  YLRSQLKQG  156-165  FTTSWNTGL  140-149  WAKKNTQGY  787-796  IAAAKGLEI  877-886  TMEAFKAIA  427-436  ALAELTPLF  579-588  FEEMVANFD  264-273  YIEDAKAAI  899-908  YFSAEDAAY  378-387  KVEDFMAKC  863-872  FMLDHFSKA  649-658  YKVTYTYSD  22-31  SRWVQKQLL  162-171  TGLAFAALI  413-422  KVEQKLHEA  233-242  FFASESKIA  807-816  FKYFDKDKS  513-522  LRERRTQFL  74-83  QKRENVDLA  906-915  AYLRSQLKQ  861-870  VRFMLDHFS  234-243  FASESKIAA  239-248  KIAAMADKI  653-662  YTYSDATGE  304-313  YNRDIRPEI | 878-887  MEAFKAIAQ  227-236  VAEFFHFFA  149-158  EHVAVNNFT  642-651  LVEFKLNYK  170-179  INKFRPNLL  800-809  LNEFKDTFK  150-159  HVAVNNFTT  228-237  AEFFHFFAS  820-829  YFELKACLT  86-95  INEVKKIRT  231-240  FHFFASESK  739-748  AEELTPIYE  643-652  VEFKLNYKV  865-874  LDHFSKAET  485-494  INEYNALAQ  866-875  DHFSKAETT | 665-674 ARLDLKQII  276-285  TVEDEKLKA  311-320  EIVDHRAKA | 317-326  AKAMRSWAA  560-569  IRNVYAVTL  820-829  YFELKACLT  783-792  IDAAIAAAK  639-648  YNELVEFKL  488-497  YNALAQPLY  900-909  FSAEDAAYL  769-778  FENLIAHID  878-887  MEAFKAIAQ  160-169  WNTGLAFAA  161-170  NTGLAFAAL  323-332  WAALVTKCK  496-505  YDEAIAFKE  291-300  IPGIRGKLA  234-243  FASESKIAA  780-789  VKEIDAAIA  604-613  SHQIPGDAA  597-606  ITALVTSSH  240-249  IAAMADKIK  150-159  HVAVNNFTT  649-658  YKVTYTYSD  875-884  ETTMEAFKA  746-755  YEDLEKDQL  131-140  ATARDALLL  787-786  IAAAKGLEI  193-202  EKAFAACKE  166-175  FAALINKFR  785-894  AAIAAAKGL  579-588  FEEMVANFD | **α-actinin** |  |

| Server | Antigen | Allele | Start | End |  | Score |
| --- | --- | --- | --- | --- | --- | --- |
| IEDB | **AP33** | HLA-DRB1*01:01 | 95 | 109 | EGKYKALEAAGVRIA | 0.9956 |
|  |  | HLA-DRB1*01:01 | 94 | 108 | AEGKYKALEAAGVRI | 0.9946 |
|  |  | HLA-DRB1*01:01 | 93 | 107 | TAEGKYKALEAAGVR | 0.9930 |
|  |  | HLA-DRB1*01:01 | 96 | 110 | GKYKALEAAGVRIAR | 0.9866 |
|  |  | HLA-DRB1*11:01 | 50 | 64 | AAEWIAKTKLTQEKP | 0.9661 |
|  |  | HLA-DRB1*11:01 | 49 | 63 | DAAEWIAKTKLTQEK | 0.9481 |
|  |  | HLA-DRB1*01:01 | 78 | 92 | NASLIFVPAPGAAAA | 0.9330 |
|  |  | HLA-DRB1*11:01 | 51 | 65 | AEWIAKTKLTQEKPV | 0.9160 |
|  |  | HLA-DRB1*01:01 | 92 | 106 | GTAEGKYKALEAAGV | 0.8888 |
|  |  | HLA-DRB1*01:01 | 63 | 77 | KPVVAFIAGATAPPG | 0.8834 |
|  |  | HLA-DRB1*11:01 | 48 | 62 | EDAAEWIAKTKLTQE | 0.8779 |
|  |  | HLA-DRB1*07:01 | 95 | 109 | EGKYKALEAAGVRIA | 0.8565 |
|  |  | HLA-DRB1*01:01 | 79 | 93 | ASLIFVPAPGAAAAC | 0.8537 |
|  |  | HLA-DRB1*08:01 | 50 | 64 | AAEWIAKTKLTQEKP | 0.8368 |
|  |  | HLA-DRB1*15:01 | 62 | 76 | EKPVVAFIAGATAPP | 0.8350 |
|  |  | HLA-DRB1*07:01 | 94 | 108 | AEGKYKALEAAGVRI | 0.8348 |
|  |  | HLA-DRB1*01:01 | 77 | 91 | ANASLIFVPAPGAAA | 0.8320 |
|  |  | HLA-DRB1*01:01 | 80 | 94 | SLIFVPAPGAAAACI | 0.8299 |
|  |  | HLA-DRB1*08:01 | 113 | 127 | QHDMIKVKKVMKETG | 0.8287 |

| Server | Antigen | Allele | Start | End |  | Score |
| --- | --- | --- | --- | --- | --- | --- |
| IEDB | **AP65** | HLA-DRB1*01:01 | 254 | 268 | FEDFEMETAFKLLDH | 0.9810 |
|  |  | HLA-DRB1*01:01 | 253 | 267 | QFEDFEMETAFKLLD | 0.9647 |
|  |  | HLA-DRB1*01:01 | 255 | 269 | EDFEMETAFKLLDHF | 0.9636 |
|  |  | HLA-DRB1*01:01 | 67 | 81 | IRRQFELMPTPLLKY | 0.9477 |
|  |  | HLA-DRB1*01:01 | 68 | 82 | RRQFELMPTPLLKYI | 0.9444 |
|  |  | HLA-DRB1*03:01 | 341 | 355 | KNIIMFDHRGMVHAG | 0.9414 |
|  |  | HLA-DRB1*08:01 | 493 | 507 | AESLASLVKKEDHDQ | 0.9326 |
|  |  | HLA-DRB1*11:01 | 52 | 66 | LLPYKVFTKDEQAAR | 0.9246 |
|  |  | HLA-DRB1*03:01 | 340 | 354 | FKNIIMFDHRGMVHA | 0.9211 |
|  |  | HLA-DRB1*08:01 | 492 | 506 | AAESLASLVKKEDHD | 0.9197 |
|  |  | HLA-DRB1*03:01 | 319 | 333 | GIANLIVDMTVSRGG | 0.9134 |
|  |  | HLA-DRB1*01:01 | 66 | 80 | RIRRQFELMPTPLLK | 0.9104 |
|  |  | HLA-DRB1*03:01 | 190 | 204 | IGQVDPDQTLPVQLD | 0.8996 |
|  |  | HLA-DRB1*11:01 | 372 | 386 | VYGSVLEAVKKFKAT | 0.8940 |
|  |  | HLA-DRB1*03:01 | 320 | 334 | IANLIVDMTVSRGGI | 0.8727 |

| Server | Antigen | Allele | Start | End |  | Score |
| --- | --- | --- | --- | --- | --- | --- |
| IEDB | **α-actinin** | HLA-DRB1*01:01 | 897 | 911 | DQYFSAEDAAYLRSQ | 0.9652 |
|  |  | HLA-DRB1*01:01 | 202 | 216 | LGIYVYLDPEDVIDT | 0.9550 |
|  |  | HLA-DRB1*01:01 | 784 | 798 | DAAIAAAKGLEISEE | 0.9407 |
|  |  | HLA-DRB1*01:01 | 896 | 910 | LDQYFSAEDAAYLRS | 0.9381 |
|  |  | HLA-DRB1*07:01 | 784 | 798 | DAAIAAAKGLEISEE | 0.9357 |
|  |  | HLA-DRB1*01:01 | 201 | 215 | ELGIYVYLDPEDVID | 0.9329 |
|  |  | HLA-DRB1*11:01 | 803 | 817 | FKDTFKYFDKDKSNS | 0.9202 |
|  |  | HLA-DRB1*13:01 | 14 | 28 | EKTQIKVFSRWVQKQ | 0.9176 |
|  |  | HLA-DRB1*07:01 | 783 | 797 | IDAAIAAAKGLEISE | 0.9176 |
|  |  | HLA-DRB1*11:01 | 804 | 818 | KDTFKYFDKDKSNSL | 0.9133 |
|  |  | HLA-DRB1*11:01 | 231 | 245 | FHFFASESKIAAMAD | 0.9067 |
|  |  | HLA-DRB1*01:01 | 203 | 217 | GIYVYLDPEDVIDTT | 0.9054 |
|  |  | HLA-DRB1*08:01 | 670 | 684 | KQIILAKKTFLEEEE | 0.9003 |
|  |  | HLA-DRB1*01:01 | 783 | 797 | IDAAIAAAKGLEISE | 0.8921 |
|  |  | HLA-DRB1*13:01 | 15 | 29 | KTQIKVFSRWVQKQL | 0.8862 |
