# Supplementary material for: Introduction of protein vaccine candidate based on AP65, AP33, and α-actinin proteins against Trichomonas vaginalis parasite: an immunoinformatics design
Source: Parasit Vectors. 2024 Mar 31;17:165. doi: 10.1186/s13071-024-06248-y (PMC10981826; doi:10.1186/s13071-024-06248-y)
Supplement: Supplementary file 3 — Additional file 3: S3. The final vaccine sequence. [file 13071_2024_6248_MOESM3_ESM.docx]

**The final vaccine sequence:**

IAFKEEVLAISGELRERRTQFLAKQAEAPTKREHVNEIDPIFDGLEKDSLHLRVNHSPTEIRNVYAVTLQHIITELNKIFE**EAAAKEAAAK**EKSVVTQVAEFFHFFASESKIAAMADKIKRTVAIQKQIDELKNTYIEDAKAAIEKMTVEDEKLKADDYEKTIPGIRGKLASVISYNRDIRPEIVDHRAKAMRSWAALVTKC**EAAAKEAAAK**EELTPIYEDLEKDQLHLEITSTPASINIFFENLIAHIDTLVKEIDAAIAAAKGLEISEEEL**EAAAKEAAAK**ENLASLDGFAEKIQALQDPYNELVEFKLNYKVTYTYSDATGELDQA**EAAAKEAAAK**KDEQAARIRRQFELMPTPLLKYIFLANEREKNSQSFWRFLFTHPPEETMPILYTPTVGEACQKWATHRQQSYRGIYITPEDSGKIKDILRNYPRQDIRCIVV**EAAAKEAAAK**IANLIVDMTVSRGGITKEQAFKNIIMFDHRGMVHAGRKDLYDFNKPYMHDM**EAAAKEAAAK**NPTPKAEATPHDVYLWSNGKALCATGSPFPAEQVNGRKVITAQANNSWIFPAVGYALVTTK**EAAAKEAAAK**LTYEAAYATTQAGLGQSTVVGIGGDPFAGQLHTDVIKRFAADPQTEGIILIGEIGGTSEEDAAEWIAKTKLTQEKPVVAFIAGATAPPGKRMGHAGAIVSGGKGTAEGKYKALEAAGVRIAR**EAAAKEAAAK**VHPKKGKIIAGLPIFKNMKEVVKRTDANASLIFVPAPGAAAACIEAAEAGMGLVVCITEHIPQHD
